# A systematic review with network meta-analysis on mono strategy of anaesthesia for preeclampsia in caesarean section

**DOI:** 10.1038/s41598-021-85179-5

**Published:** 2021-03-11

**Authors:** Chu Cheng, Alan Hsi-Wen Liao, Chien-Yu Chen, Yu-Cih Lin, Yi-No Kang

**Affiliations:** 1 School of Medicine, College of Medicine, Taipei Medical University, Taipei, Taiwan; 2 Department of Anesthesiology, Taipei Medical University Hospital, No. 252, Wuxing Street, Taipei, 11031 Taiwan; 3 Department of Anesthesiology, School of Medicine, College of Medicine, Taipei Medical University, Taipei, Taiwan; 4 School of Nursing, College of Nursing, Taipei Medical University, Taipei, Taiwan; 5 Evidence-Based Medicine Center, Wan Fang Hospital, Taipei Medical University, Taipei, Taiwan; 6 Research Center of Big Data and Meta-Analysis, Wan Fang Hospital, Taipei Medical University, Taipei, Taiwan; 7 Cochrane Taiwan, Taipei Medical University, Taipei, Taiwan; 8 Institute of Health Policy and Management, College of Public Health, National Taiwan University, Taipei, Taiwan

**Keywords:** Health care, Medical research

## Abstract

The aim of this study was to reveal the effects of anaesthesia strategies on maternal mean arterial pressure (MAP), heart rate, vasopressor consumption, adverse events, and neonatal resuscitation when women with preeclampsia (PE) undergo caesarean section (CS). Three major databases were searched for randomized controlled trials (RCTs) and prospective controlled studies (PCSs). Two authors independently screened, extracted, and checked eligibility and outcome data. Outcomes involved MAP, vasopressor use, maternal adverse events, APGAR scores, and neonatal resuscitation. Pooled estimates were carried out by contrast-based network meta-analysis, and pooled effect sizes were presented with 95% confidence interval (CI). Eleven RCTs and one PCS (n = 782) formed three-node network meta-analysis, and non-significant differences were observed in MAP, 5-min APGAR score, and neonatal intubation rate among the three anaesthesia strategies. General anaesthesia had significantly lower vasopressor consumption than spinal anaesthesia did (standardised mean difference =  − 1.19, 95% confidence interval [CI]: − 1.76 to − 0.63), but it had higher maternal adverse event rate (risk ratio = 2.00, 95% CI 1.16–3.47). Because no optimal anaesthesia strategy has been shown to achieve a balanced maternal and neonatal outcome, therefore a shared decision-making process may be required regarding the most suitable choice of anaesthetic strategy for individual preeclamptic mother undergoing CS. Future larger studies may need to focus on evaluating the role of vasopressors on maternal hemodynamic as well as factors affecting maternal outcomes for different anaesthetic techniques in preeclamptic women undergoing CS.

## Introduction

Preeclampsia (PE), a multisystem disease during pregnancy, is of concern to both anaesthesiologists and gynaecologists. This is because preeclamptic patients are susceptible to cerebrovascular, cardiopulmonary, renal, and haematological events, and foetal outcomes might be compromised because of the associated poor placental function^1,2^. Although the prevalence of PE differs by gestational age, a systematic review revealed that the prevalence of PE is approximately 4.6% (95% uncertainty range: 2.7–8.2%) of pregnancies worldwide^3^. Controversy has surrounded the optimal anaesthesia strategy for preeclamptic patients when they undergo caesarean section (CS) for many years. Until 2015, trial and review studies have investigated the most suitable anaesthetic technique for preeclamptic patients; however, a systematic analysis of their data is lacking. Although a network meta-analysis compared anaesthesia strategies for CS, it did not specified women with preeclampsia^4^. Moreover, the network meta-analysis admitted that pooling preeclampsia with normal women would be inappropriate due to heterogeneity. A synthesis comparing anaesthesia strategies for preeclampsia in CS is still needed.

Both general anaesthesia and regional anaesthesia (spinal and epidural anaesthesia) have advantages and disadvantages. General anaesthesia is often thought to be unsafe because of several maternal airway-related factors, such as possible difficult airways, failed intubation, and hypertensive response to laryngoscopy and intubation^5^. However, under certain circumstances, general anaesthesia may be indicated for emergent CS, such as in cases that require a reassuring airway and for patients with severe coagulopathy^6^. By contrast, reports have claimed that spinal anaesthesia (SA) is associated with less hypotension and less impairment of cardiac function in preeclamptic patients compared with that in healthy patients^7,8^. Therefore regional anaesthesia has been accepted by many as the superior anaesthetic technique for CS in preeclamptic patients because it appears to have fewer associated complications. Furthermore, it has provided superior analgesia compared with general anaesthesia, resulting in less circulating catecholamine and possible improvement in placental blood flow^9–12^. Nevertheless, other clinicians have discouraged SA over the concern that it may cause profound hypotension and worsen new born outcomes.

Epidural anaesthesia (EA) offers the advantages of a more stable hemodynamic profile, lower fluid requirements, and lower neuroendocrine stress compared with general anaesthesia^13,14^. Furthermore, some studies have shown that, in contrast to SA, EA does not produce sudden hypotension in patients with PE. Recent studies, however, have revealed that the haemodynamic effect of EA is equivalent to that of SA^15–17^. Nevertheless, concerns remain regarding regional anaesthesia in patients with PE because severe PE or Haemolysis, Elevated Liver enzymes, Low Platelet count (HELLP) syndrome may lead to the development of thrombocytopenia, which increases the risk of spinal or epidural haematoma. EA should not be used for patients with a platelet count less than 80 × 10^9^ L^−1^^18^, because the use of larger needles might induce massive haemorrhage^19,20^.

Therefore, selecting the optimal anaesthesia strategy for preeclamptic patients is difficult because many clinical points must be considered. The purpose of this study was to conduct a meta-analysis to determine the best available evidence to guide clinical practice.

## Results

**Figure 1:**
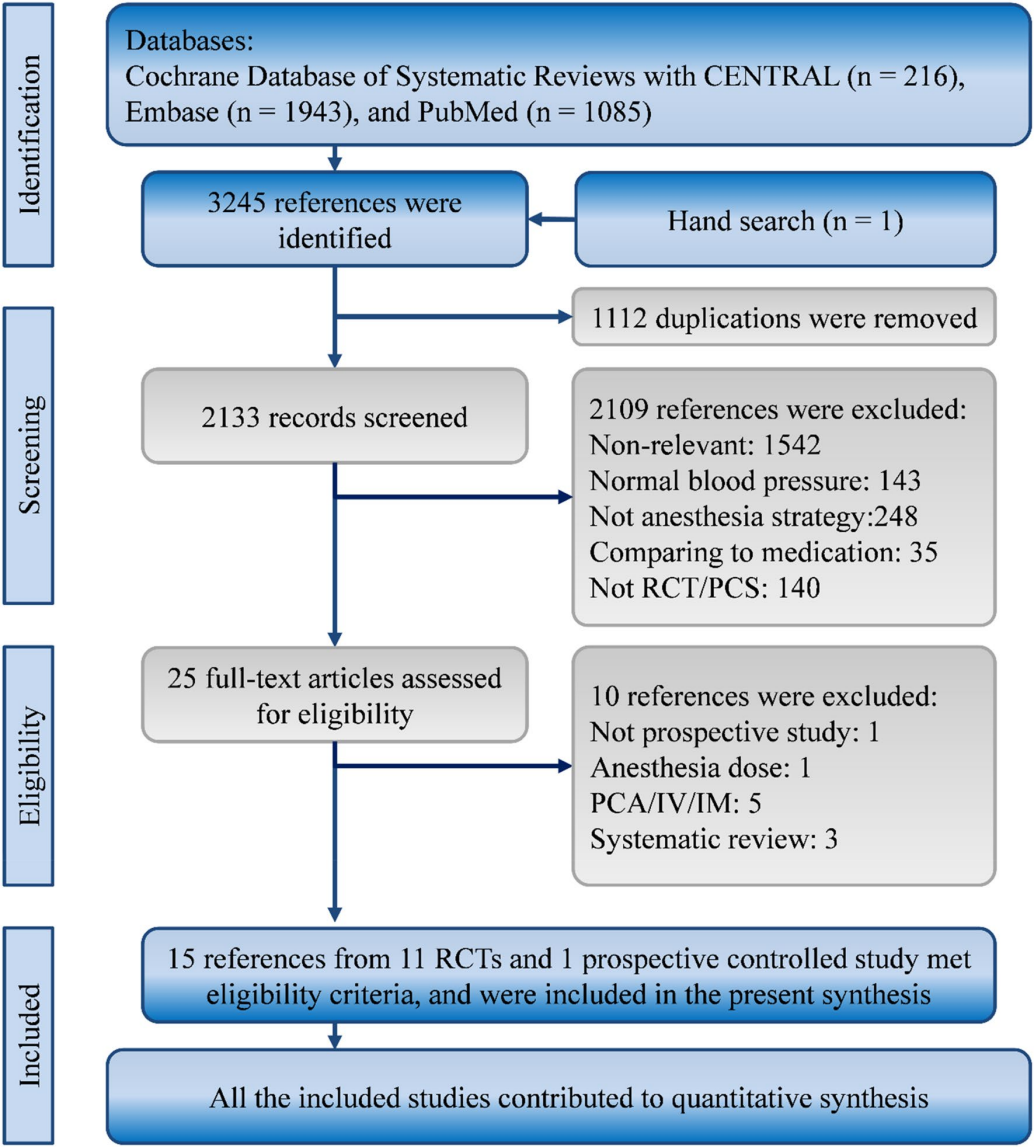
Flow diagram of study selection. *IM* intramuscular, *IV* intravenous, *PCA* patient-controlled analgesia, *PCS* prospective controlled study, *RCT* randomized controlled trial.

Our search strategy yielded 3244 studies from the Cochrane Database of Systematic Reviews and the CENTRAL (k=216), Embase (k=1943), and New PubMed (k=1085) databases. Moreover, a further study was obtained by hand. After we removed 1112 duplicate and 2109 irrelevant studies through title and abstract screening, we accessed the full texts of the remaining 25 studies and carefully reviewed all the texts. Fifteen articles from 11 randomized controlled trials (RCTs) and a prospective controlled study met the eligibility criteria, and we included all of them in our synthesis (Fig. 1)^8,13,16,21–31^.

### Characteristics and quality of included studies

**Figure 2:**
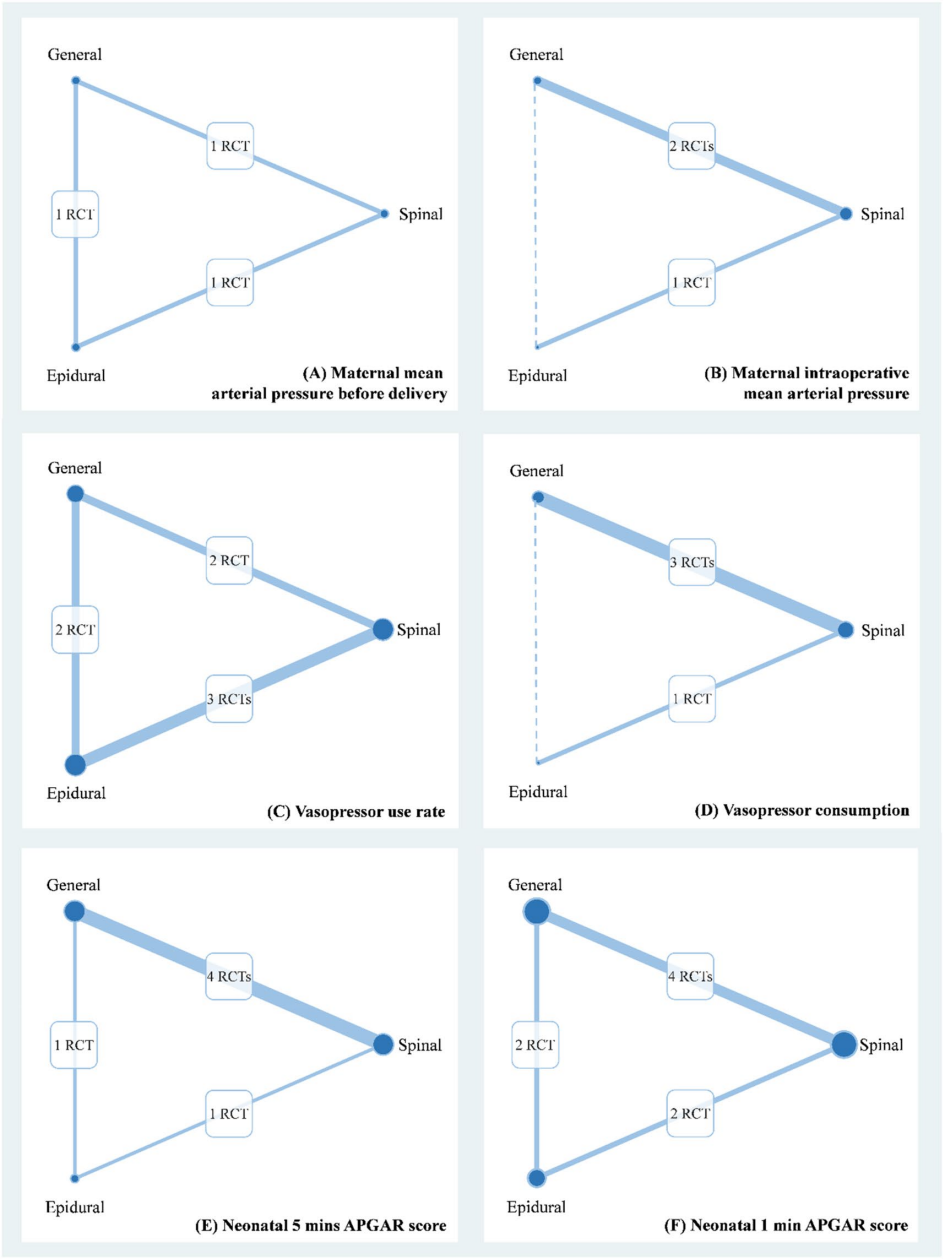
Network plot of anaesthesia strategies for (**A**) maternal mean arterial pressure before delivery, (**B**) maternal intraoperative mean arterial pressure, (**C**) vasopressor consumption, (**D**) vasopressor use rate, (**E**) neonatal 5 min APGAR score, and (**F**) neonatal 1 min APGAR score.

The 11 RCTs and a prospective nonrandomized controlled trial recruited a total of 782 women with PE from Canada^29^, India^21,22,32^, Iran^26^, the Republic of Macedonia^28^, Russia^23–25^, South Africa^8^, Thailand^16^, the United Kingdom^27^, and the United States^13,30,31^ between 1980 and 2015. The mean age of the women ranged from 18.8 to 32 years. Table 1 presents relevant information about maternal mean arterial pressure (MAP) at baseline, gestational age, and baby body weight; furthermore, Appendix 1 shows the quality of the RCTs and a prospective controlled study. These trials formed three-node network for primary outcomes (Fig. 2).

**Table 1:** Characteristics of the included clinical trials.

Author	Location	Anaesthesia	Cases	Mean	MAP	GA	Baby body
		strategy		Age			Weight (g)
Hodgkinson	Canada	General	10	22.9	Unclear	NR	2709.3
1980		Epidural	10	22.3	Unclear	NR	2395.9
Ramanathan	United	General	11	18.8	133	33.2	2229
1991	States	Epidural	10	19.3	133	32.7	2216
Wallace	United	General	26	NR	123.89	34	2138
1995	States	Epidural	27	NR	120.54	34	2158
Sharwood	United	Spinal	11	29.7	NR	33.8	NR
1999	Kingdom	Epidural	10	27.3	NR	35	NR
Mathur	India	Spinal	21	24.9	Unclear	NR	2020
2002		General	20	25.9	Unclear	NR	2700
		Epidural	20	25.3	Unclear	NR	2740
Dyer	South	Spinal	35	25	121	34.9	2138
2003	Africa	General	35	26	120	35.1	2236
Visalyaputra	Thailand	Spinal	47	30	136	36	2410
2005		General	53	32	138	37	2401
Moslemi	Iran	Spinal	30	30.17	114.99	34–39	NR
2007		General	30	28.40	115.72	34–39	NR
Dasgupta	India	Spinal	41	23.11	129.98	38.49	NR
2011		General	41	21.9	130.69	37.67	NR
Kinzhalova	Russia	Spinal	30	27.03	117.55	32.58	1658
(Central) 2012		General	30	28.19	116.89	33.23	1680
Chattopadhyay	India	Spinal	147	23.42	NR	35.8	2480
2014		General	27	22.78	NR	34.63	2280
Sivevski	Republic of	Spinal	30	25	120.4	34.8	1400
2015	Macedonia	General	30	26	117.4	34.9	1500

*GA* gestational age, *MAP* mean artery pressure, *NR* no report.

### Maternal cardiovascular conditions

**Figure 3:**
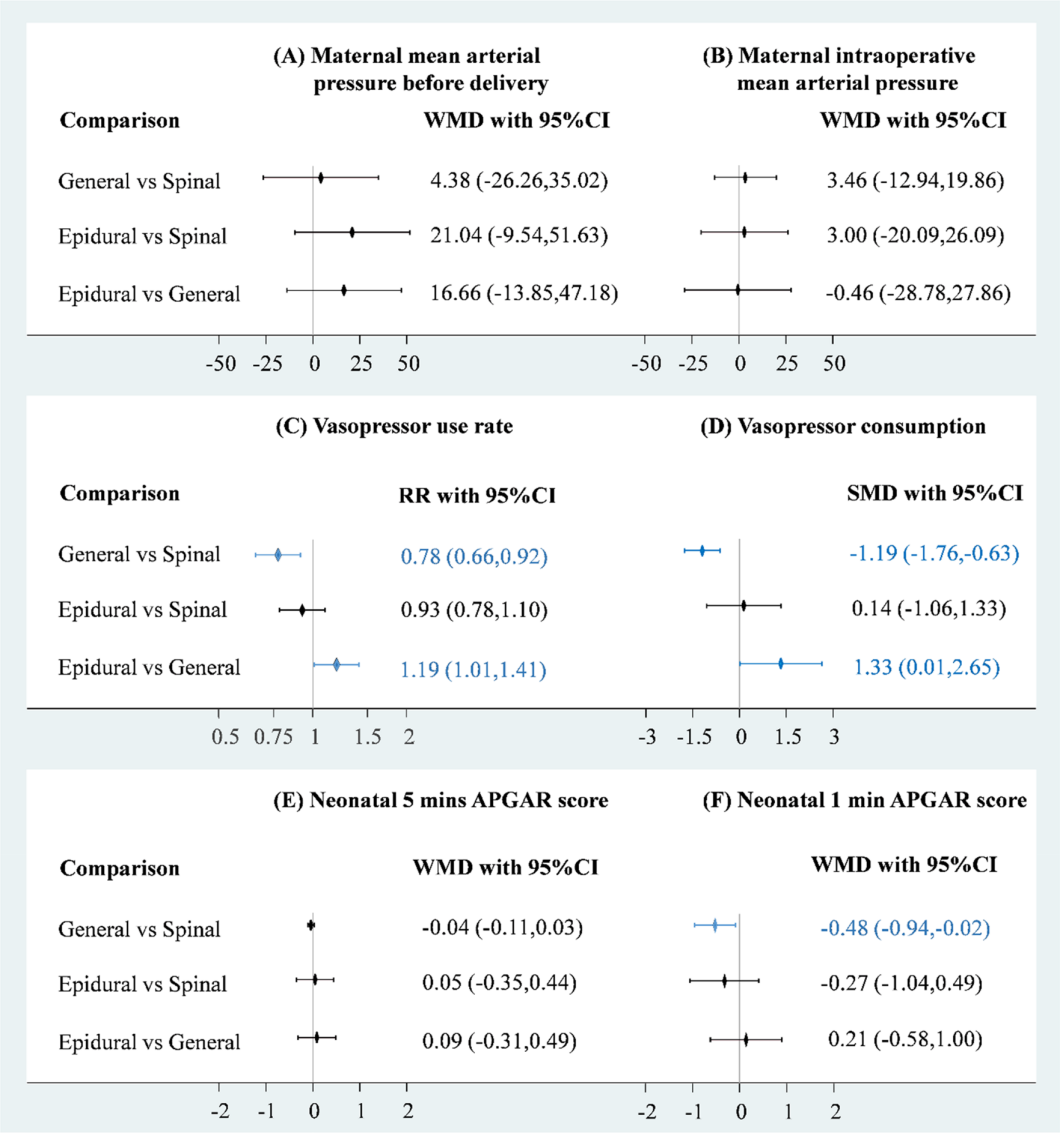
Forest plot of anaesthesia strategies for (**A**) maternal mean arterial pressure before delivery, (**B**) maternal intraoperative mean arterial pressure, (**C**) vasopressor consumption, (**D**) vasopressor use rate, (**E**) neonatal 5 min APGAR score, and (**F**) neonatal 1 min APGAR score.

Maternal cardiovascular conditions involved MAP, heart rate (HR), systemic vascular resistance (SVR), vasopressor consumption, and vasopressor use rate. Data on HR and SVR were only available from the studies with comparisons of general anaesthesia and SA. Consequently, HR and SVR could only be pooled in a head-to-head meta-analysis. A total of four RCTs (n=251) reported MAP before delivery or intraoperative (Fig. 2A,B)^8,13,16,23–25^. Pooled estimates revealed no significant difference in MAP before delivery or intraoperative among the three anaesthesia strategies (Fig. 3A,B); however, weighted mean difference (WMD) in MAP before delivery between EA and the other anaesthesia strategies raised clinical concerns. The WMD between EA and SA was 21.04 and that between EA and general anaesthesia was 16.66. Therefore, we further performed cumulative probability ranking with 10 000 repetitions, and the results indicated that EA had the highest probability of leading to higher MAP (81.1%; Appendix 2). Loop inconsistency raised some concerns in the pooled estimate of MAP before delivery (chi-square=38.43, *P*<0.05; Appendix 3), but the funnel plot with Egger’s test did not indicate asymmetry or a significant small study effect in the network meta-analysis of MAP before delivery and intraoperative (Appendix 4 and 5).

A total of two RCTs (n = 130) reported data regarding the mean HR before delivery and intraoperative^8,23–25^. However, these studies only compared general anaesthesia with SA. Based on the available data, patients who received SA had lower HR than those who received general anaesthesia before delivery (WMD = − 31.70, 95% confidence interval [CI] − 39.98 to − 23.42) and intraoperative (WMD = − 16.48, 95% CI − 21.38 to − 11.58). Although the pooled estimates were based on small-scale RCTs (Appendix 6), the included studies consistently exhibited lower HR trends in SA (I-square = 0%). Data on SVR were only available from an RCT with three references^23–25^. Patients who received SA had significantly lower SVR before delivery compared with those who received general anaesthesia, but no difference existed in mean SVR intraoperative between the two anaesthesia strategies (Appendix 7).

### Vasopressor use and maternal safety

Vasopressor use rate and vasopressor consumption were presented in seven of the included studies (n = 620)^8,16,21,22,27,28,30–32^. Pooled results revealed that patients who received general anaesthesia seemed to be associated with lower risk of vasopressor use compared with those who received SA (risk ratio [RR]= 0.78, 95% CI 0.66–0.92) and EA (RR= 0.84, 95% CI 0.71–0.99), while there was no significant difference between EA and SA (Fig. 3C). Furthermore, no evidence was detected of inconsistency (chi-square=1.06, *P *> 0.10) (Appendix 8) or small study effect (coefficient = 1.80, *P *> 0.10) in the pooled estimate of vasopressor use rate (Appendix 9). Patients who received general anaesthesia was associated with lower vasopressor consumption compared with those who received SA (standardised mean difference [SMD] = − 1.19, 95% CI − 1.76 to − 0.63) and EA (SMD = − 1.33, 95% CI − 2.65 to − 0.01) (Fig. 3D). Loop inconsistency was not tested for because no loop existed in the comparison between the three anaesthesia strategies. Moreover, no evidence of severe small study effects was detected (coefficient = − 0.41, *P*>0.05) in the pooled estimate of vasopressor consumption (Appendix 10).

In addition, data on adverse events were reported in five studies (n=331)^21,26,27,29,30^. Three of them compared general anaesthesia with SA^21,26^, one of them compared SA with EA^27^, and the other two studies compared general anaesthesia with EA (Appendix 11)^29,30^. Pooled estimates showed that general anaesthesia had significantly higher adverse event than SA did (RR = 2.00, 95% CI 1.16–3.47), but no difference in maternal adverse event existed in other comparisons (Appendix 12). Furthermore, no evidence existed for loop inconsistency (chi-square = 0.16, *P *> 0.05; Appendix 13) or small study effects (coefficient = 0.62, *P *> 0.05; Appendix 14).

### Neonatal outcomes

Although appearance, pulse, grimace, activity, and respiration (APGAR) score and resuscitation are two critical neonatal outcomes during delivery, only seven of the included studies reported APGAR scores (n = 479)^21,22,25–27,29,32^. Five of them compared general anaesthesia with SA^21,22,26,32^, two of them compared SA with EA^27,32^, and other studies compared general anaesthesia with EA^29,32^. Both of 5-min APGAR scores (Fig. 2E) and 1-min APGAR scores (Fig. 2F) were contributed by six RCTs. Only one significant difference could be observed among the estimates for APGAR scores, and babies in general anaesthesia group had significantly lower 1-min APGAR score as compared with those in SA group (Weighted mean difference = − 0.48, 95% CI − 0.94 to − 0.02) (Fig. 3E,F). The Lu–Ades method did not reveal significant loop inconsistency in the pooled estimates of 5-min APGAR scores (chi-square = 0.49, *P *> 0.05; Appendix 15) and design-by-treatment interaction model did not show significant inconsistency in the result of 1-min APGAR scores (chi-square = 0.80, *P *> 0.10; Appendix 16). Moreover, no evidence indicated small study effects in the pooled estimates of 5-min APGAR scores (coefficient = − 0.95, *P* > 0.05; Appendix 17) or 1-min APGAR scores (coefficient = − 0.76, *P *> 0.05; Appendix 18).

**Figure 4:**
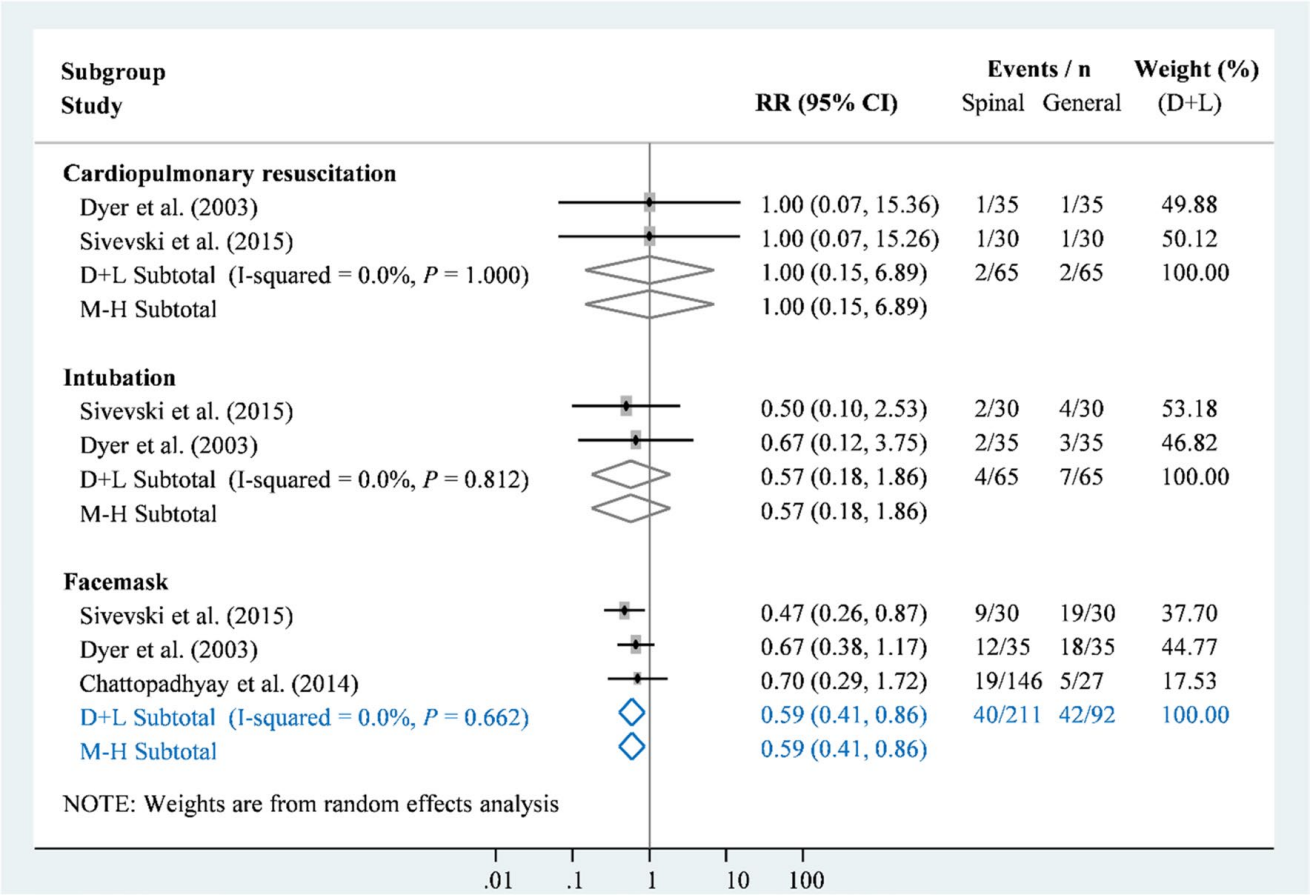
Forest plot of anaesthesia strategies for neonatal resuscitation.

Neonatal resuscitations were mainly reported in three studies with comparisons of general and SA (n = 303)^8,21,28^, and only one trial with a comparison of SA and EA reported neonatal intubation (Appendix 19)^30^. Based on the available data, no significant difference was observed in neonatal intubation between the three anaesthesia strategies (Appendix 20 to 21). However, SA had a significantly lower rate of facemask use than did general anaesthesia (RR = 0.59, 95% CI 0.41–0.86; Figure 4). This result also had very low heterogeneity (I-square = 0%, *P*>0.10).

## Discussion

For preeclamptic patients, neuraxial anaesthesia (SA and EA) has been considered the optimal choice for CS due to its advantages of attenuation of hypertensive response and superior pain relief. Because of concerns over difficult airway management, coagulopathy, postpartum haemorrhage, renal failure, the need for postoperative intensive care, and postoperative complications, many reports have recommended against general anaesthesia for patients undergoing CS^21,33–35^. However, our findings did not support these recommendations, and post hoc evaluation of confidence in the findings of our meta-analysis were shown in Table 2. Some studies have revealed that general anaesthesia does not pose higher risks to neonates, yet other studies have indicated that general anaesthesia may be associated with adverse neonatal outcomes, higher rates of low 1- and 5-min APGAR scores, a greater requirement for advanced resuscitation, and even mortality^21,33–35^.

**Table 2:** Assessing confidence in results of network meta-analysis.

Comparison	Number of studies	Within-study bias	Reporting bias	Indirectness	Imprecision	Heterogeneity	Incoherence	Confidence rating
**Maternal mean arterial pressure before delivery**								
Epidural vs General	1	Some concerns	Undetected	No concerns	Major concerns	–^b^	Major concerns	Very low
Epidural vs Spinal	1	Some concerns	Undetected	No concerns	Major concerns	–^b^	Major concerns	Very low
General vs Spinal	1	Some concerns	Undetected	No concerns	Major concerns	–^b^	Major concerns	Very low
**Maternal intraoperative mean arterial pressure**								
Epidural vs General	0	–^a^	–^a^	Major concerns	Some concerns	–^a^	–^c^	Very low
Epidural vs Spinal	1	Some concerns	Undetected	No concerns	Some concerns	–^b^	No concerns	Low
General vs Spinal	2	Some concerns	Undetected	No concerns	Some concerns	No concerns	No concerns	Moderate
**Vasopressor use rate**								
Epidural vs General	2	Some concerns	Undetected	No concerns	No concerns	No concerns	No concerns	Moderate
Epidural vs Spinal	3	Some concerns	Undetected	No concerns	No concerns	Major concerns	No concerns	Low
General vs Spinal	2	Some concerns	Undetected	No concerns	No concerns	No concerns	No concerns	Moderate
**Vasopressor consumption**								
Epidural vs General	0	–^a^	–^a^	Major concerns	Some concerns	–^a^	–^c^	Very low
Epidural vs Spinal	1	Some concerns	Undetected	No concerns	Some concerns	–^b^	No concerns	Low
General vs Spinal	3	Some concerns	Undetected	No concerns	No concerns	Some concerns	No concerns	Low
**Overall adverse event**								
Epidural vs General	2	Some concerns	Undetected	No concerns	Some concerns	No concerns	No concerns	Moderate
Epidural vs Spinal	1	Some concerns	Undetected	No concerns	Some concerns	–	No concerns	Moderate
General vs Spinal	2	Some concerns	Undetected	No concerns	Some concerns	No concerns	No concerns	Moderate
**Neonatal resuscitation**								
Epidural vs General	1	Some concerns	Undetected	No concerns	Major concerns	–^b^	No concerns	Very low
Epidural vs Spinal	0	–^a^	–^a^	Major concern	Major concerns	–^a^	–^c^	Very low
General vs Spinal	3	Some concerns	Undetected	No concerns	Major concerns	Some concerns	No concerns	Very low
**Neonatal 5 min APGAR score**								
Epidural vs General	1	Some concerns	Undetected	No concerns	Some concerns	–^b^	No concerns	Low
Epidural vs Spinal	1	Some concerns	Undetected	No concerns	Some concerns	–^b^	No concerns	Low
General vs Spinal	4	Some concerns	Undetected	No concerns	No concerns	Major concerns	No concerns	Low
**Neonatal 1 min APGAR score**								
Epidural vs General	2	Some concerns	Undetected	No concerns	Some concerns	No concerns	No concerns	Low
Epidural vs Spinal	2	Some concerns	Undetected	No concerns	Some concerns	No concerns	No concerns	Low
General vs Spinal	4	Some concerns	Undetected	No concerns	No concerns	Some concerns	No concerns	Low

^a^Paucity of direct comparison.

^b^Single trial.

^c^No closed loop.

### Maternal outcomes

According to our data set, we found that SA led to more frequent use of vasopressors than did general anaesthesia, but it had relatively more stable maternal HR and maternal SVR. By contrast, EA seemed to have a more stable MAP. Notably, general anaesthesia did not result in less favourable haemodynamic parameters, including MAP and SVR; the overall complication rate was slightly higher but it did not reach statistical significance. During the intraoperative period, no type of anaesthesia exhibited clinical or statistical differences in terms of MAP or SVR, which was probably because of the appropriate intraoperative management steps taken, including the use of vasopressors and fluid administration.

Current evidence has suggested that maternal blood pressure should be controlled within 160/110 mmHg^36–43^. Furthermore, the blood pressure of preeclamptic patients during the perioperative period, especially the intraoperative period, should be maintained close to their baseline blood pressure instead of a textbook normal blood pressure. To prevent severe systolic hypertension with subsequent loss of cerebral vasculature autoregulation, diastolic and systolic blood pressures are recommended to be controlled within 90–105 and 140–155 mm Hg, respectively; alternatively, MAP should be controlled between 105 and 125 mm Hg^44^. Based on the primary articles, in patients receiving general anaesthesia, the MAP before foetal delivery was higher than the ideal MAP. By contrast, the MAP before foetal delivery of the patients receiving SA was closer to the lower limit of the ideal MAP range, and some even had a MAP less than the suggested MAP range following the phase of anaesthetic induction^16^. This might explain why preeclamptic patients under SA required more frequent use of vasopressors as well as larger does. One prospective study^45^, however, indicated that preeclamptic patients had less hypotension than patients without PE during SA. Nevertheless, to avoid hypotension under SA, vasopressors are often necessary for preeclamptic patients when their MAP is less than 60 mmHg or if their blood pressure decreases by more than 10%.

Pregnancy induces several maternal physiological and biochemical adjustments to ensure sufficient delivery of oxygen and nutrients to the foetus through the placenta. To compensate for SVR decreasing, HR usually increases by 20%, and the maternal cardiac output increases by 30% to 40% for the purpose of maintaining an adequate placenta perfusion^46^. PE is characterised by a pathological increase of systemic vascular resistance in response to endogenous angiotensin II and catecholamine secretions^47^. According to our primary studies, the SVR of patients receiving SA was significantly lower than that of patients receiving general anaesthesia between the time of anaesthetic induction to foetal delivery; however, no SVR differences existed during the rest of the intraoperative period. We postulated that this was because of the use of vasopressors, and evidently from our analysis, the rate of the use and dosage of vasopressors were higher in the SA group.

Vasopressors are known to be used to increase the vascular tone through constricting blood vessels, leading to an elevated SVR and increased MAP. Some vasopressors also demonstrate inotropic and chronotropic properties, resulting in both raised HR and cardiac contractility^48,49^. In our study, the greater use of vasopressors in the SA group might have increased maternal cardiac output and SVR through increased cardiac contractility and HR, which would explain the lower SVR fluctuation during most of the intraoperative period.

Collectively, SA might not be the ideal anaesthetic strategy because it causes less haemodynamic stability through the rapid onset of sympathectomy, which may result in severe hypotension. With fine-tuned coordination between obstetricians, anaesthetists, and paediatricians, general anaesthesia may provide a shorter anaesthetic time, which might accelerate foetal delivery—and this would ultimately provide the relief for maternal hypertension. As for the effect of EA on maternal outcomes, the lack of data and primary articles may have rendered the analysis nonsignificant.

### Neonatal safety

An ideal anaesthetic technique for CS should have the fewest side effects, such as haemodynamic compromise and neonatal depression. Controversy exists concerning the choice of anaesthetic technique for CS in patients with PE. And according to our analysis, the choice may not be as relevant as no differences were observed in the 5-min APGAR scores, which is known to predict the requirement for neonatal resuscitation. We further divided neonatal resuscitation into three parts, namely mask ventilation, intubation, and full CPR for comparison purposes Comparing general anaesthesia with SA, only the demand for mask ventilation was significantly higher in the general anaesthesia group. The reason for this is unknown as the details about the indication for mask ventilation were lacking in the primary articles. Furthermore, no significant difference was observed for advanced resuscitation between the anaesthesia groups.

Neonatal mortality is another crucial outcome to consider. However, remarkably few studies have recorded neonatal mortality. Moreover, mortality cannot be appropriately pooled because of insufficient data in the articles and issues such as foetuses with extremely low body weight (<1500 g) and hydrops foetalis. Currently, the effect of anaesthetic strategy on neonatal mortality is difficult to measure or estimate.

## Limitations

Our study had several limitations. First, our synthesis had a limited sample size (n = 721), and a complete network meta-analysis could only be performed for maternal MAP, vasopressor use rate, maternal adverse events, and APGAR scores. Results of HR, SVR, and neonatal resuscitation were only based on limited comparisons of the three anaesthesia strategies. The limited number of trials in the present synthesis may result in unrobust and underpowered estimates. Second, no further related studies have been conducted since 2015. With only one small-scale trial (n = 60) from the Republic of Macedonia that compared general anaesthesia and SA, we may not fully evaluate the effect of recent anaesthetic advances on the outcomes of our study. Third, wide CIs appeared in the estimates of MAP and CPR. The wide CIs may be due to the nature of wide range of MAP in patients with hypertension and small sample size with rare events of CPR. Increasing the study sample size is necessary for studies in the future. Fourth, poor methodologies in our primary studies may have contributed to the overall bias and affected the results of our study.

## Conclusions

This is the first network meta-analysis to provide an overview of the maternal and foetal effects of different mono-strategy of anaesthesia for women with PE undergoing CS. No single anaesthetic strategy was found to be superior in terms of overall neonatal outcome. When comparing spinal and general anaesthesia, spinal anaesthesia was found to be associated with lower heart rate, SVR and more vasopressor use, yet general anaesthesia was found to have higher maternal adverse events and neonatal facemask use. A shared decision-making process may be required regarding the most suitable choice of anaesthetic strategy for individual preeclamptic mother undergoing CS. Future larger studies may need to focus on evaluating the role of vasopressors on maternal hemodynamic as well as factors affecting maternal outcomes for different anaesthetic techniques in preeclamptic women undergoing CS.

## Methods

In this study, we adhered to the Cochrane Handbook for Systematic Reviews of Interventions and the PRISMA guidelines for conducting and reporting meta-analyses^50,51^. Thus, the present study did not approach patients or any human. The answerable question for this study was structured according to the following PICO framework:

Population: patients with PE who underwent CS.

Intervention: general anaesthesia.

Comparison: SA or EA.

Outcomes: maternal cardiovascular status; maternal adverse events; neonatal resuscitation; and APGAR score.

To comprehensively understand the effects of anaesthesia strategies on patients with PE undergoing CS, we not only applied head-to-head meta-analysis but also constructed a contrast-based consistency model to pool quantitative data. Protocol of this study had been registered on PROSPERO before we initiated this synthesis in 2019, and the registry number is CRD42020152390.

### Eligibility criteria and evidence selection

Two authors (C.C. and Y.N.K.) defined selection criteria according to the proposed PICO framework prior to a comprehensive search, and selected evidence if a study (a) recruited patients with PE undergoing CS, (b) compared different anaesthesia strategies, and (c) was a RCT or prospective nonrandomised clinical study with two or more groups. Because PE, CS, and anaesthesia were the three core elements of this study, we searched the Cochrane Database of Systematic Reviews (including Cochrane Central Register of Controlled Trials, CENTRAL), Embase, and the New PubMed using terms related to PE, CS, and anaesthesia in free text, medical subject headings (MeSH or Emtree), and abbreviations. In this search strategy, we did not restrict language or date of publication. The final search was done for reference before September, 2020 (Appendix 22).

The study selection process involved two steps: (a) title and abstract screening and (b) full-text review. The two authors (C.C. and Y.C.L.) independently excluded studies after screening titles and abstracts. Then, they retrieved the full texts of the potential studies for an extensive review. In addition to the abovementioned primary criteria, they further removed studies according to the following exclusion criteria: (a) studies that recruited healthy pregnant women and patients with PE without conducting a stratified analysis; (b) studies that mainly compared anaesthesia regimens; (c) studies that mainly compared anaesthesia doses; (d) studies with no comparison between anaesthesia strategies; and (e) grey literature without details about study designs or outcomes. An experienced researcher (Y.N.K.) made the final decisions if the first two authors had disagreed in the study selection process. Flowchart of selection was illustrated using Microsoft PowerPoint.

### Data extraction and quality assessment

The two authors also individually extracted data regarding study information, baseline characteristics, and outcomes. Study information included study location, recruitment duration, allocation method, blinding, loss to follow-up, type of data analysis, sponsors, and conflicts of interest. Baseline characteristics were anaesthesia strategy, mean age, gestational age, baseline HR, and baseline blood pressure. Outcomes were maternal HR, maternal MAP, maternal SVR, maternal vasopressor use, maternal adverse events, neonatal resuscitation, and 1-min and 5-min APGAR scores. The two authors extracted the number of cases and total cases for maternal vasopressor use, maternal adverse events, and neonatal resuscitation because these outcomes are commonly presented as rates. Furthermore, they determined the means and standard deviations, along with the total sample size, for maternal HR, maternal MAP, maternal SVR, maternal vasopressor use, and 1-min and 5-min APGAR scores because these were continuous variables. Based on the study information, the two authors assessed the quality of the RCTs using the Cochrane Risk of Bias Tool.

### Data synthesis and analysis

Quantitative synthesis in this study mainly relied on contrast-based network meta-analysis and head-to-head meta-analysis. RRs were calculated for maternal vasopressor use, maternal adverse events, and neonatal resuscitation. RD was also calculated when the RR indicated clinical concern (0.5 or 2) without significance. WMD was estimated for maternal HR, maternal MAP, maternal SVR, and 1-min and 5-min APGAR scores. Because vasopressors could vary between trials, we used SMD for maternal vasopressor use. In the results, we present effect size (RR, RD, WMD, or SMD) with the 95% CI. Heterogeneity in the pooled results was examined using the I-square statistics. An estimate was considered highly heterogeneous when the I-square statistic was higher than 50% or the *P* value of the heterogeneity test was higher than 0.10. Funnel plots and Egger’s test were also used to reveal small study effects. When the p value of Egger’s test is lower than 0.05, the pooled estimate may be affected by a small study effect. We further employed surface under the cumulative ranking curve (SUCRA) to numerically present the overall ranking for each anaesthesia strategy. By ranking the probability of each anaesthesia strategy in terms of efficacy, the SUCRA illustrated echelons for identifying the optimal strategy. In addition, we tested inconsistency in each network meta-analysis by using the inconsistency test, which is a method for reflecting transitivity and consistency^52–54^. Inconsistency test was mainly done using loop inconsistency method by Lu and Ade^55^. Inconsistency test for vasopressor use rate and 1 minute APGAR score was based on design-by-treatment interaction model since these outcomes consisted of two-arm and three-arm RCTs^56^. Pooled results should be interpreted with care when high heterogeneity, a small study effect, or inconsistencies are detected in the synthesis. Network meta-analysis was done using “network” package^57^, and head-to-head meta-analysis was carried out by “metan” package^58^. The “network” package is based on packages “mvmeta,” “metareg,” and “networkplot”^57^. These packages can be run in STATA version 14 for Microsoft Windows (Texas, USA). Network plots were generated by STATA, and further polished in Microsoft PowerPoint. Overall quality of results of network meta-analysis were further evaluated for clinical implication confidence based on the concept of relevant rules^59^.

## Supplementary Information

Supplementary Information 1.

Supplementary Information 2.

## Data Availability

All data generated or analysed during this study are included in this published article.

## Abbreviations

APGAR: Appearance, pulse, grimace, activity, and respiration
CI: Confidence intervals
HR: Heart rate
MAP: Mean arterial pressure
PE: Preeclampsia
RCT: Randomized clinical trial
RR: Risk ratio
SMD: Standardized mean difference
SUCRA: Surface under the cumulative ranking
SVR: Systemic vascular resistance
WMD: Weighted mean difference
