# Supplementary material for: A systematic review with network meta-analysis on mono strategy of anaesthesia for preeclampsia in caesarean section
Source: Sci Rep. 2021 Mar 11;11:5630. doi: 10.1038/s41598-021-85179-5 (PMC7970950; doi:10.1038/s41598-021-85179-5)
Supplement: Supplementary file 1 — Supplementary Information 1. [file 41598_2021_85179_MOESM1_ESM.pdf]

APPENDIX

Appendix 1. Quality of the included randomized controlled trials

First author's family name	Randomization	Allocation	Blinding	Loss-follow up	Type of analysis	Selective Report	Other source of bias
Hodgkinson 1980	Unclear (no details)	Unclear (no details)	No blinding	0	ITT	Low risk	Low risk
Ramanathan 1991	Unclear (no details)	Unclear (no details)	No blinding	0	ITT	Low risk	Low risk
Wallace 1995	Random table	Sealed envelope	No blinding	0	ITT	Low risk	Low risk
Sharwood 1999	Unclear (no details)	Unclear (no details)	No blinding	25%	ITT	Low risk	Low risk
Mathur 2002	Unclear (no details)	Unclear (no details)	No blinding	0	ITT	Low risk	Low risk
Dyer 2003	Unclear (no details)	Sealed envelope	No blinding	0	ITT	Low risk	Low risk
Visalyaputra 2005	Random table	Sealed envelope	No blinding	4.2%	ITT	Low risk	Low risk
Moslemi 2007	Unclear (no details)	Unclear (no details)	No blinding	0	ITT	Low risk	Low risk
Dasgupta 2011	Unclear (no details)	Sealed envelope	No blinding	4.8%	ITT	Low risk	Low risk
Kinzhalova 2012	Unclear (no details)	Envelope	No blinding	0	ITT	Low risk	Low risk
Chattopadhyay 2014	Unclear (no details)	Unclear (no details)	No blinding	0	ITT	Low risk	Low risk
Sivevski 2015	Unclear (no details)	Unclear (no details)	No blinding	0	ITT	Low risk	Low risk

Appendix 1. Quality of the included randomized controlled trials

First author's family name	Selection bias	Performance bias	Detection bias	Attrition bias	Reporting bias	Other source of bias
Hodgkinson 1980	Unclear	High risk	High risk	Low risk	Low risk	Low risk
Ramanathan 1991	Unclear	High risk	High risk	Low risk	Low risk	Low risk
Wallace 1995	Low risk	High risk	High risk	Low risk	Low risk	Low risk
Sharwood 1999	Unclear	High risk	High risk	High risk	Low risk	Low risk
Mathur 2002	Unclear	High risk	High risk	Low risk	Low risk	Low risk
Dyer 2003	Unclear	High risk	High risk	Low risk	Low risk	Low risk
Visalyaputra 2005	Low risk	High risk	High risk	Low risk	Low risk	Low risk
Moslemi 2007	Unclear	High risk	High risk	Low risk	Low risk	Low risk
Dasgupta 2011	Unclear	High risk	High risk	Low risk	Low risk	Low risk
Kinzhalova 2012	Unclear	High risk	High risk	Low risk	Low risk	Low risk
Chattopadhyay 2014	Unclear	High risk	High risk	Low risk	Low risk	Low risk
Sivevski 2015	Unclear	High risk	High risk	Low risk	Low risk	Low risk

Appendix 2. Cumulative probability ranking of MAP before delivery

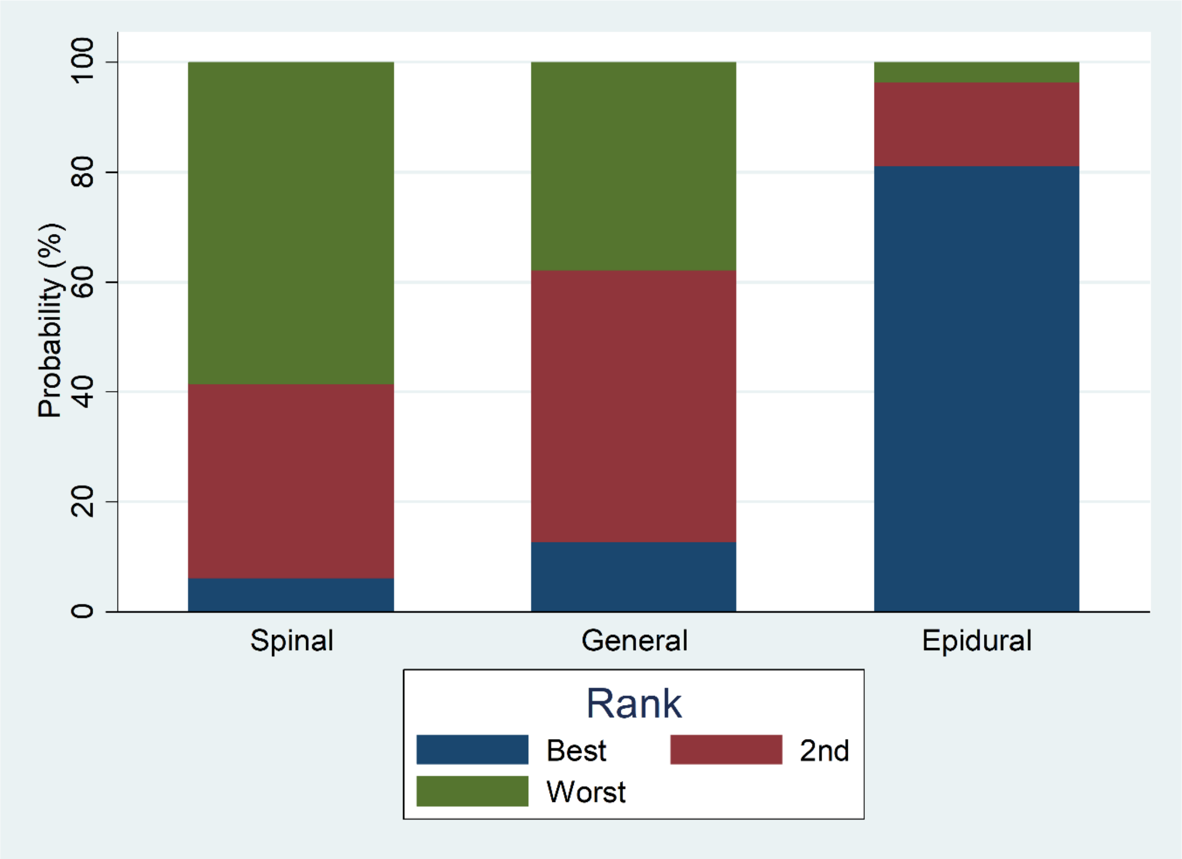

Treatment Relative Ranking of Model 1

Treatm~t	SUCRA	PrBest	MeanRank
Spinal	23.8	6.2	2.5
General	37.5	12.7	2.3
Epidural	88.7	81.1	1.2

Appendix 3. Loop inconsistency test of MAP before delivery

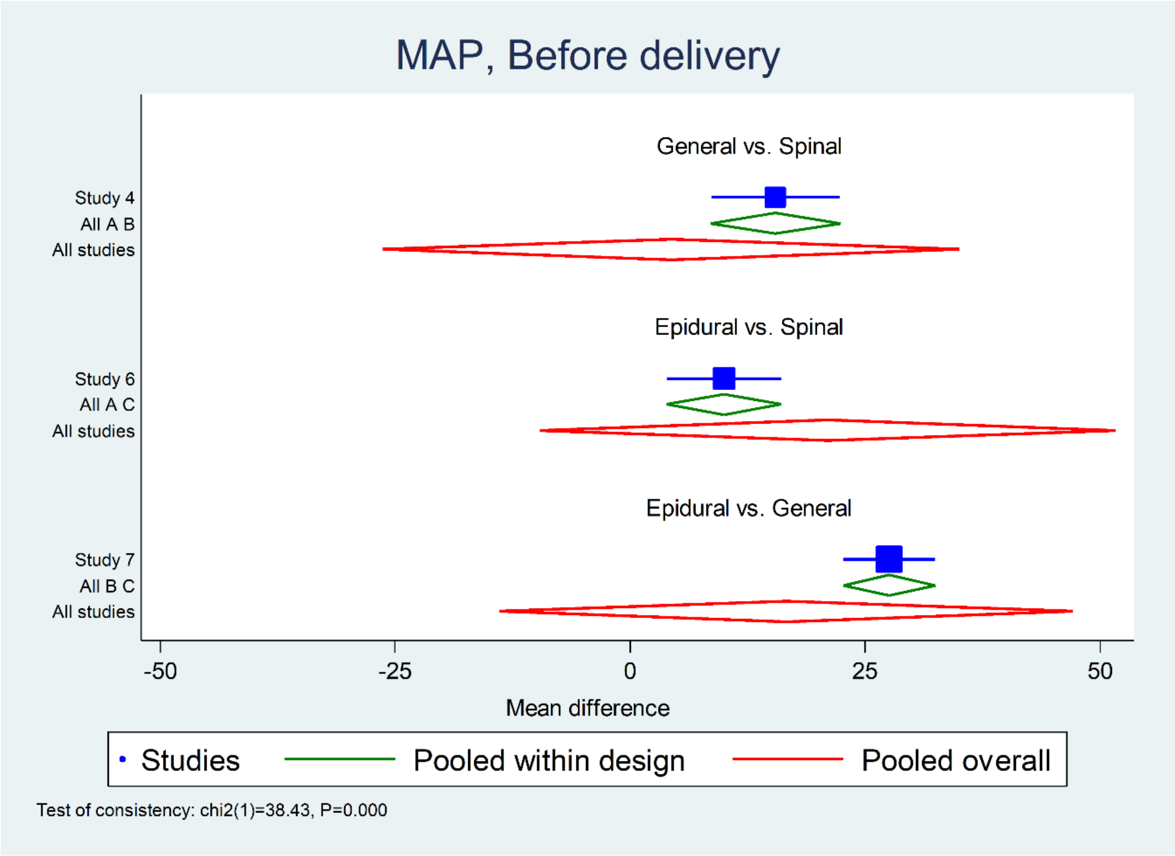

Appendix 4. Publication bias test of MAP before delivery

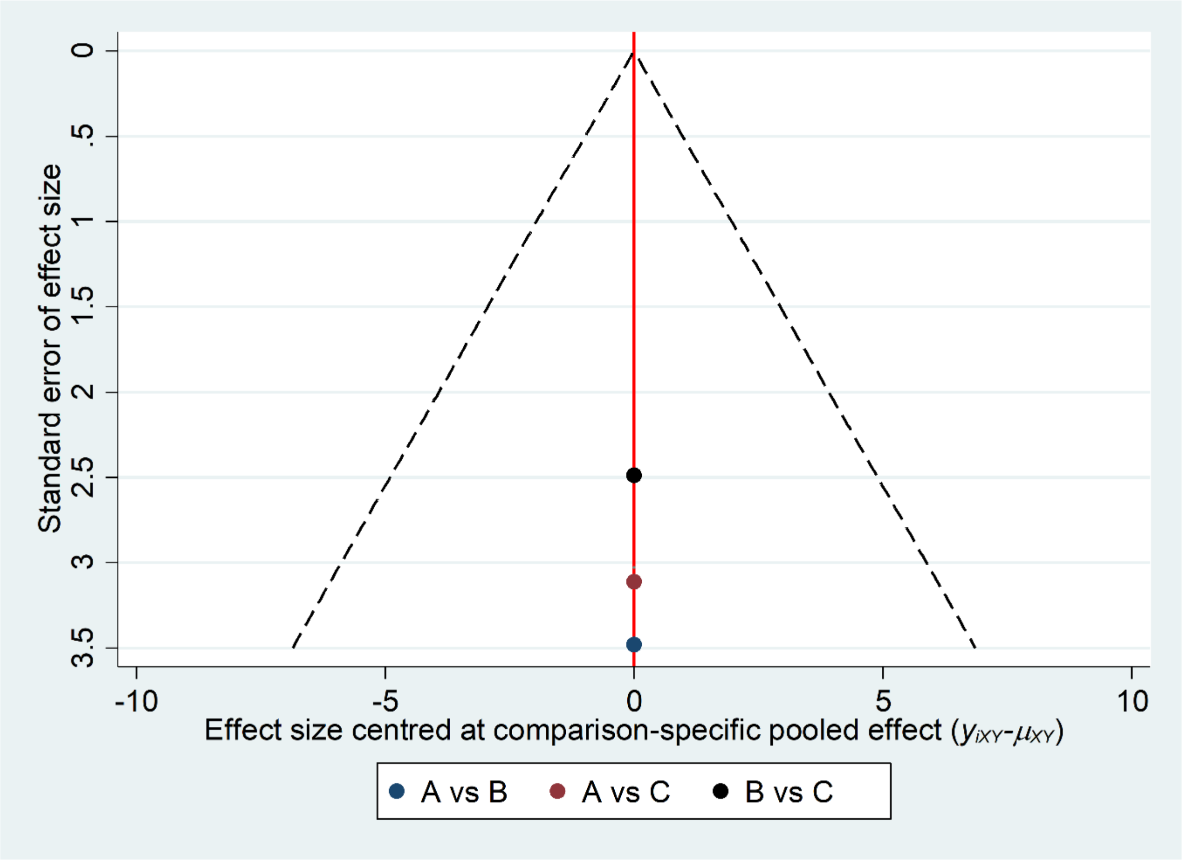

Appendix 5. Publication bias test of intraoperative MAP

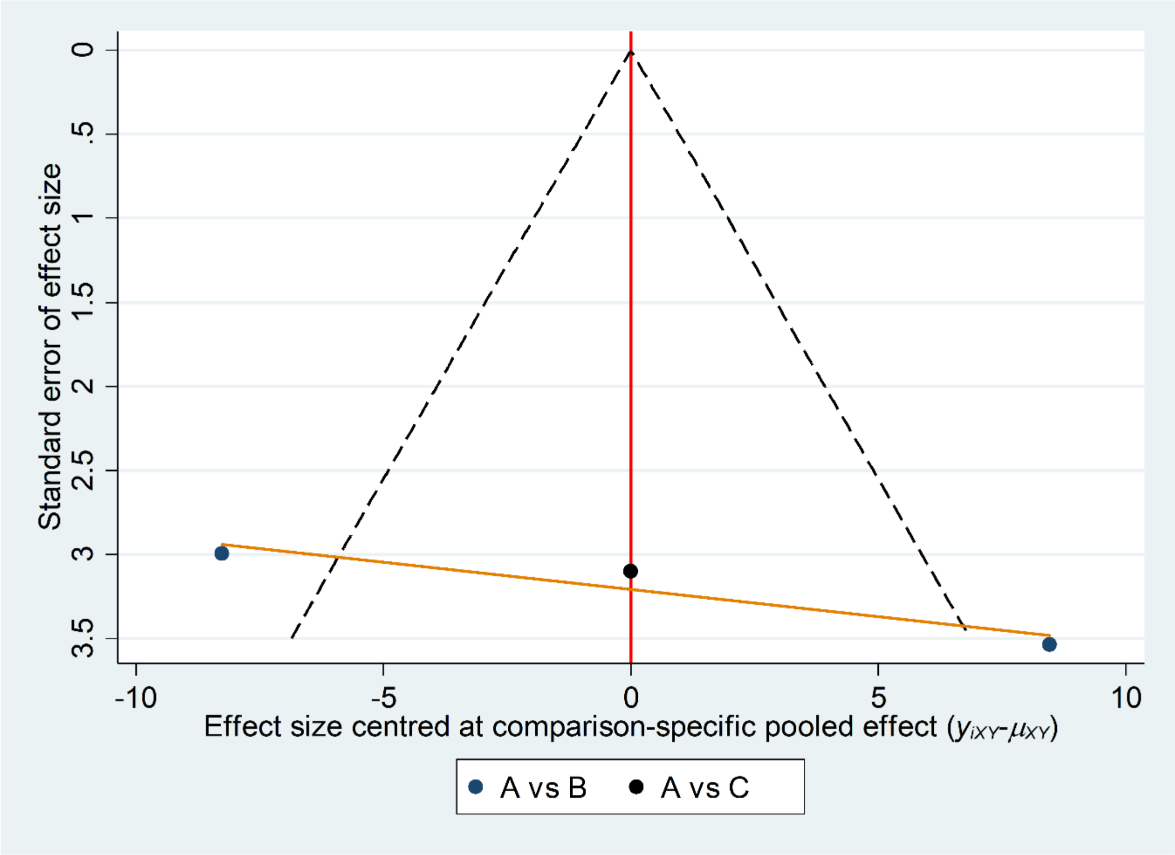

Egger's test for small-study effects:

Number of studies = 3 Root MSE = 1.25

Std_Eff	Coef.	Std. Err.	t	P>|t|	[95% Conf.	Interval]
slope	-90.23121	32.91961	-2.74	0.223	-508.5145	328.0521
bias	28.12358	10.32982	2.72	0.224	-103.1293	159.3764

Test of H0: no small-study effects P = 0.224

Appendix 6. Forest plot of maternal heart rate

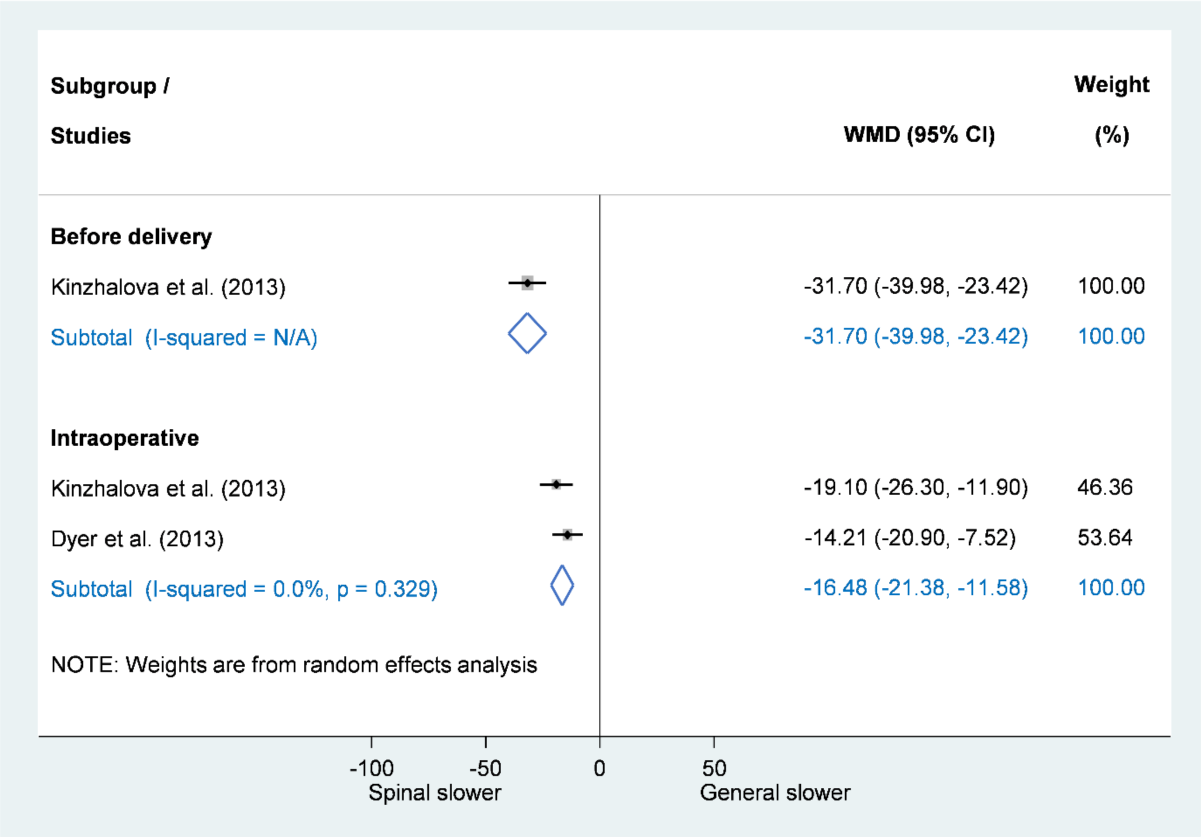

Appendix 7. Forest plot of maternal systemic vascular resistance

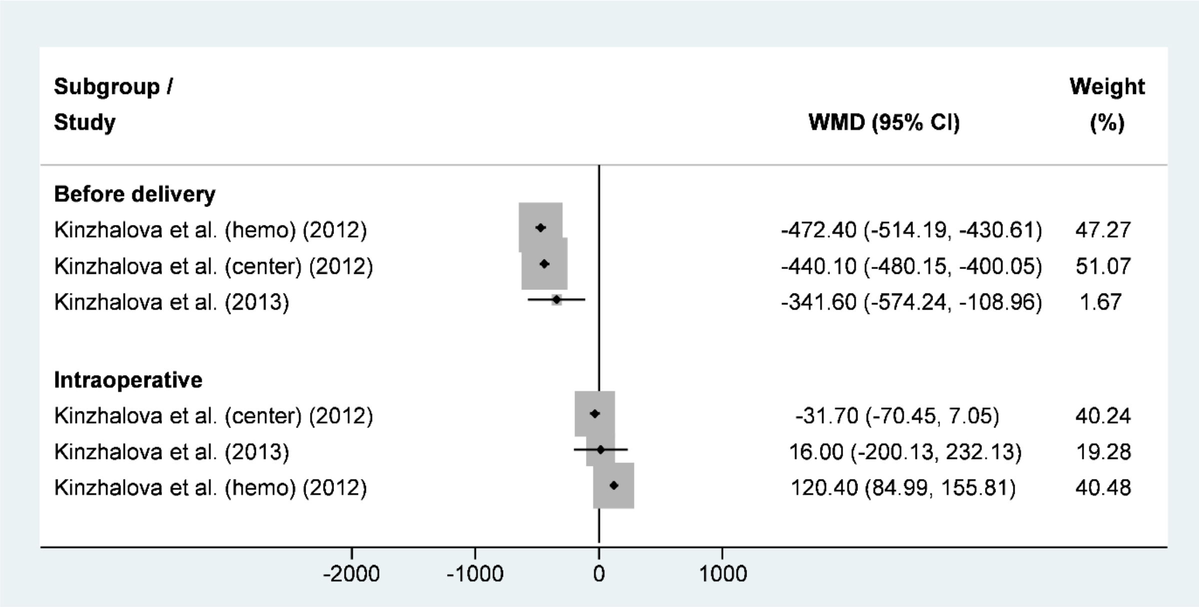

Appendix 8. Inconsistency test of vasopressor usage rate

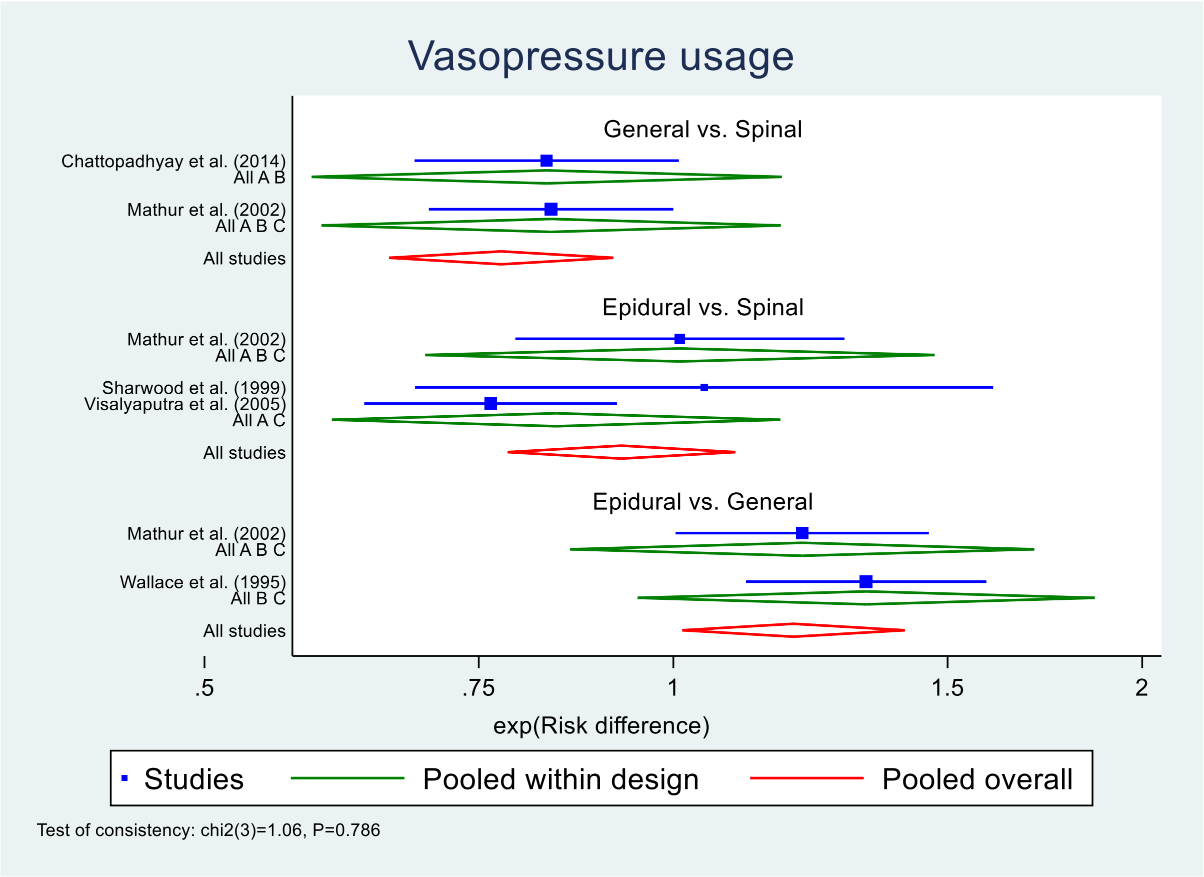

Appendix 9. Publication bias test of vasopressor usage rate

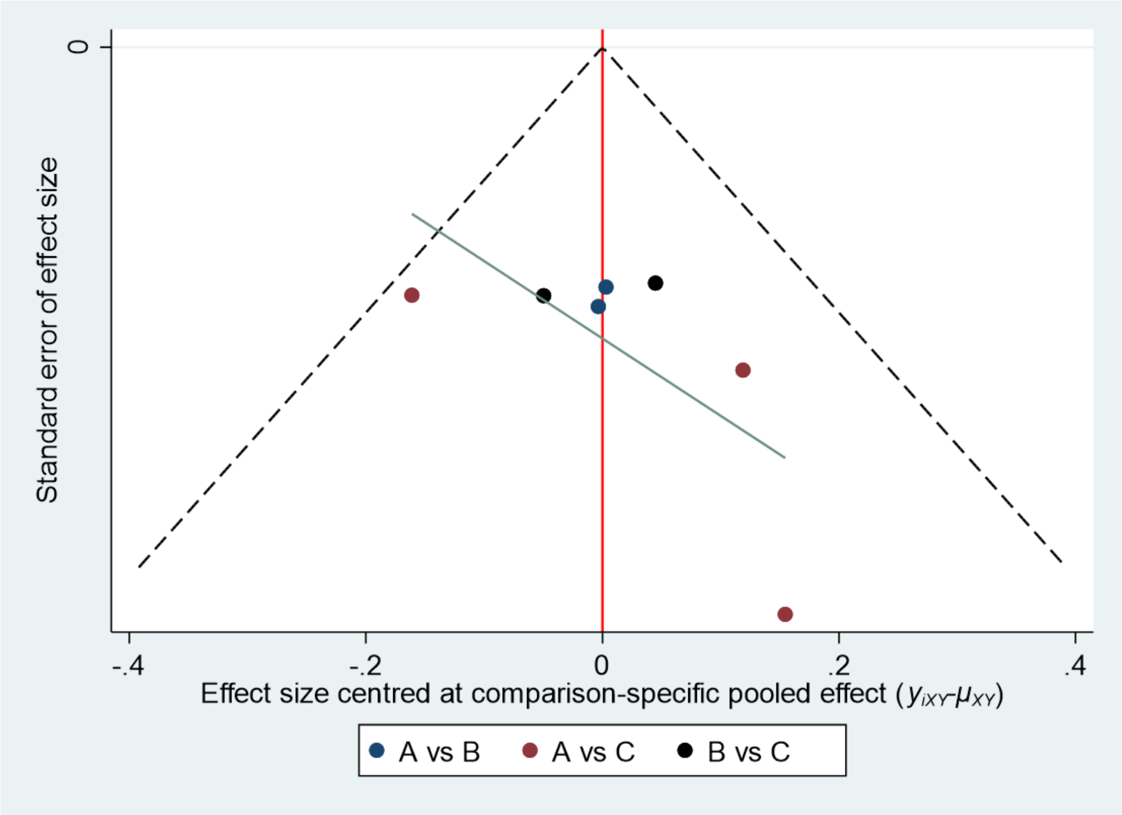

Number of studies = 7 Root MSE = .8461

Std_Eff	Coef.	Std. Err.	t	P>|t|	[95% Conf.	Interval]
slope	-.1933366	.1485781	-1.30	0.250	-.5752686	.1885955
bias	1.801457	1.426623	1.26	0.262	-1.865795	5.468709

Test of H0: no small-study effects P = 0.262

Appendix 10. Publication bias test of vasopressor consumption

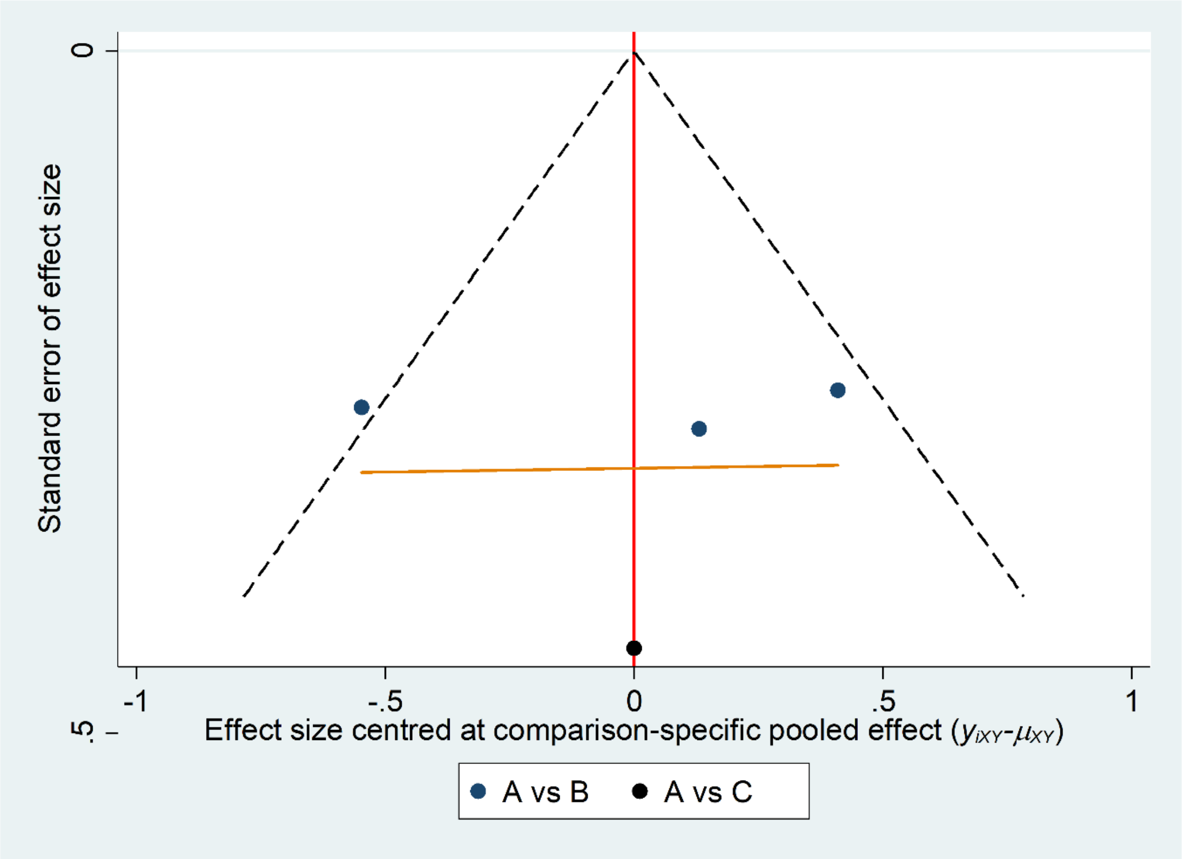

Egger's test for small-study effects:

Number of studies = 4 Root MSE = 1.913

Std_Eff	Coef.	Std. Err.	t	P>|t|	[95% Conf.	Interval]
slope	.1210136	1.404481	0.09	0.939	-5.92198	6.164007
bias	-.4095028	4.921714	-0.08	0.941	-21.58593	20.76692

Test of H0: no small-study effects P = 0.941

Appendix 11. Network plot of maternal adverse event

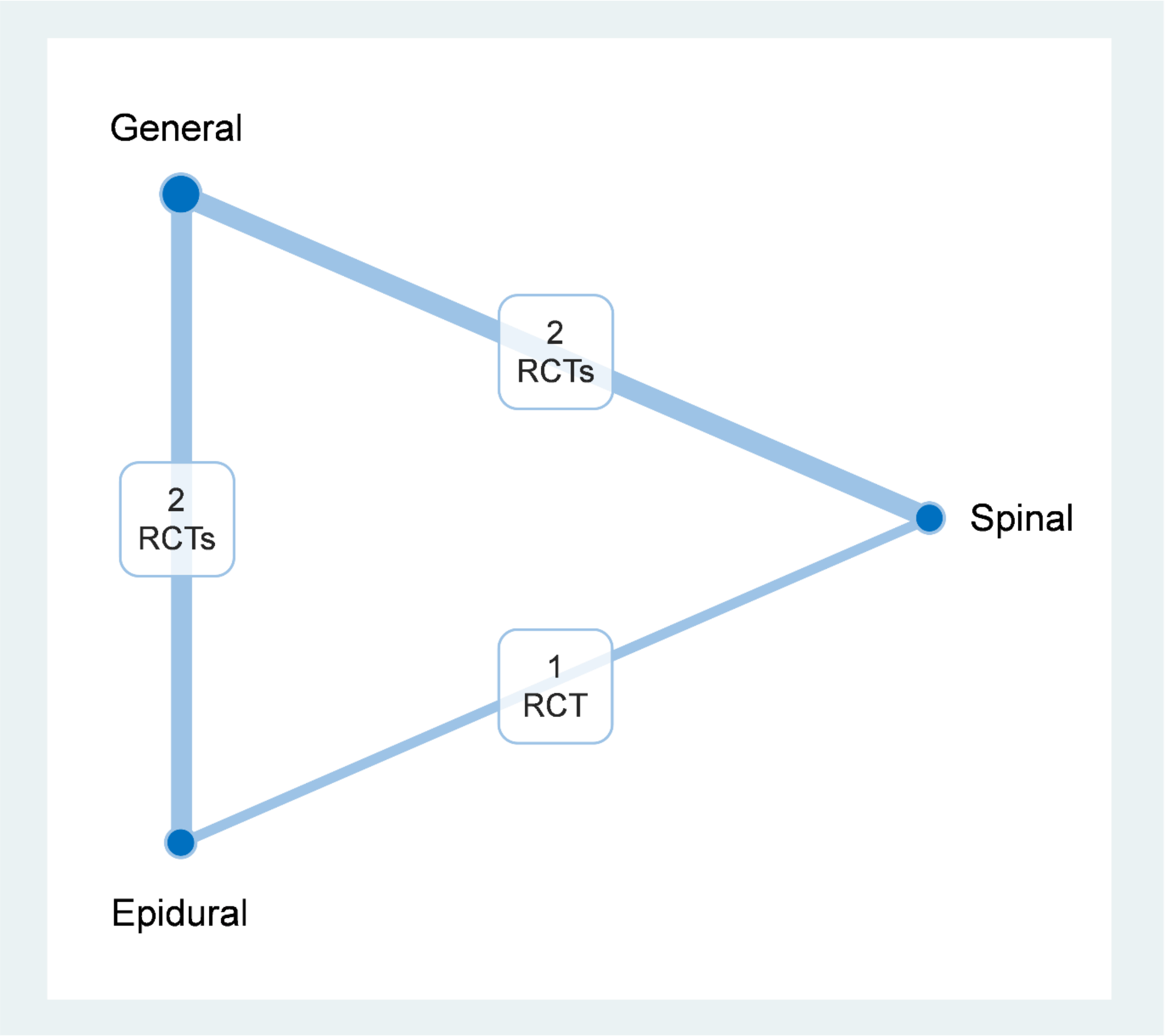

Appendix 12. Forest plot of maternal adverse event

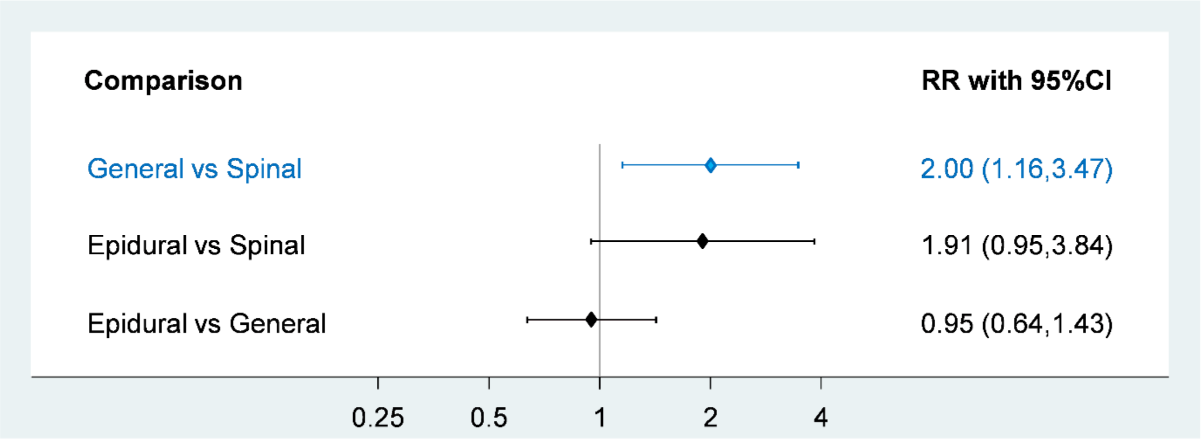

Appendix 13. Loop inconsistency test of maternal adverse event

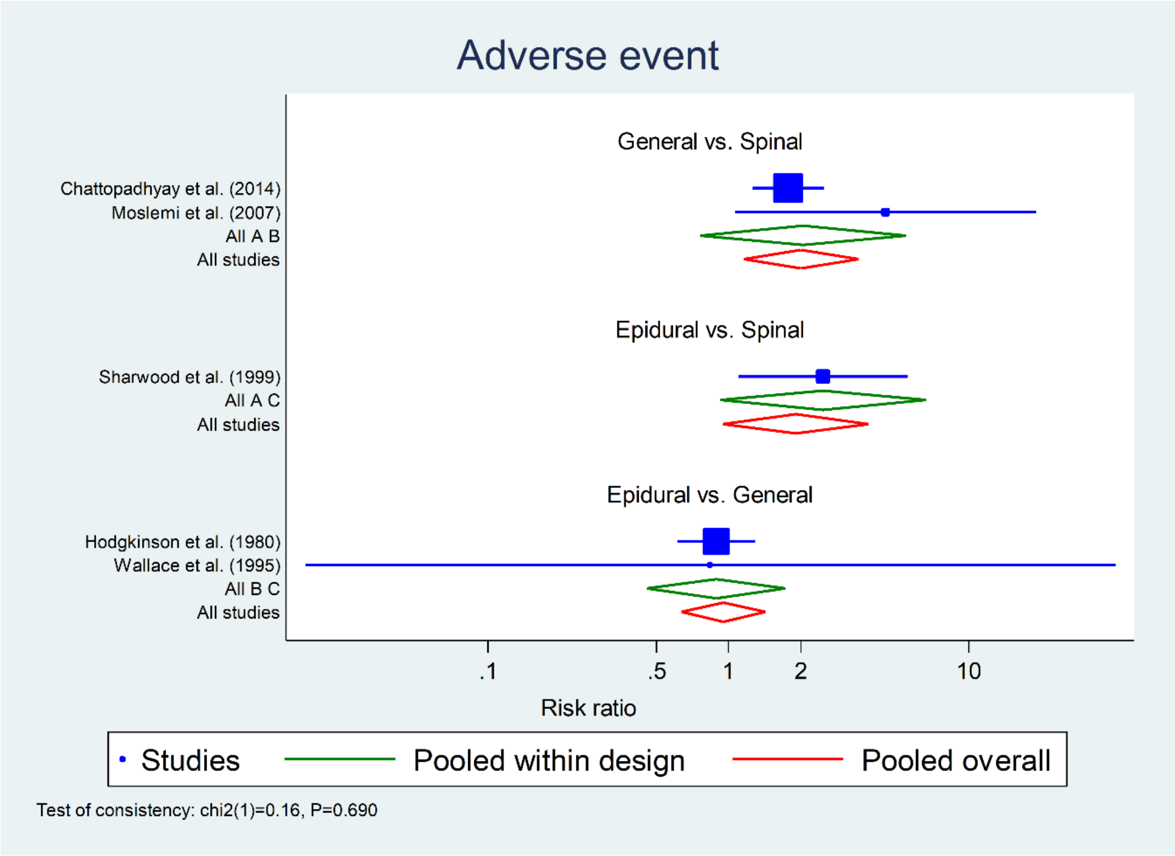

Appendix 14. Publication bias test of maternal adverse event

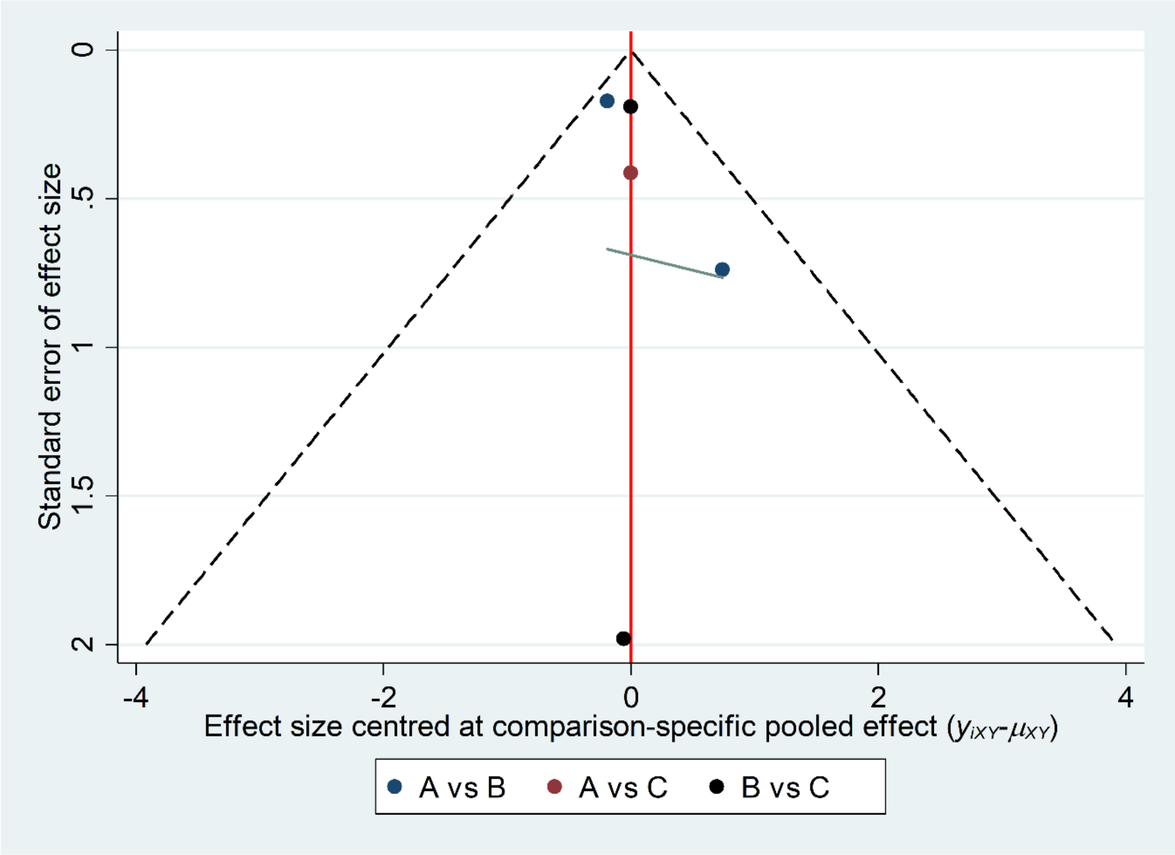

Egger's test for small-study effects:

Number of studies = 5 Root MSE = .6468

Std_Eff	Coef.	Std. Err.	t	P>|t|	[95% Conf.	Interval]
slope	-.2100287	.1374729	-1.53	0.224	-.6475288	.2274714
bias	.6190405	.5112758	1.21	0.313	-1.008067	2.246148

Test of H0: no small-study effects P = 0.313

Appendix 15. Loop inconsistency test of 5-min APGAR scores

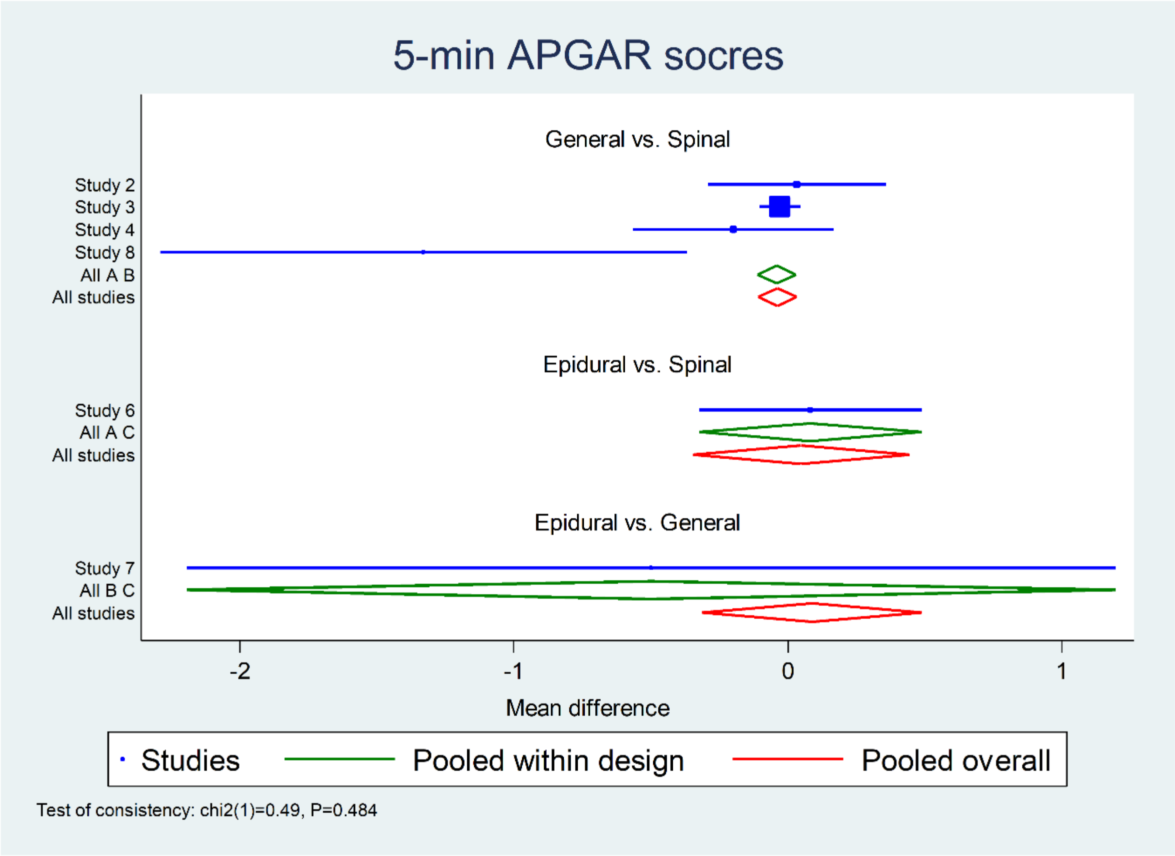

Appendix 16. Loop inconsistency test of 1-min APGAR scores

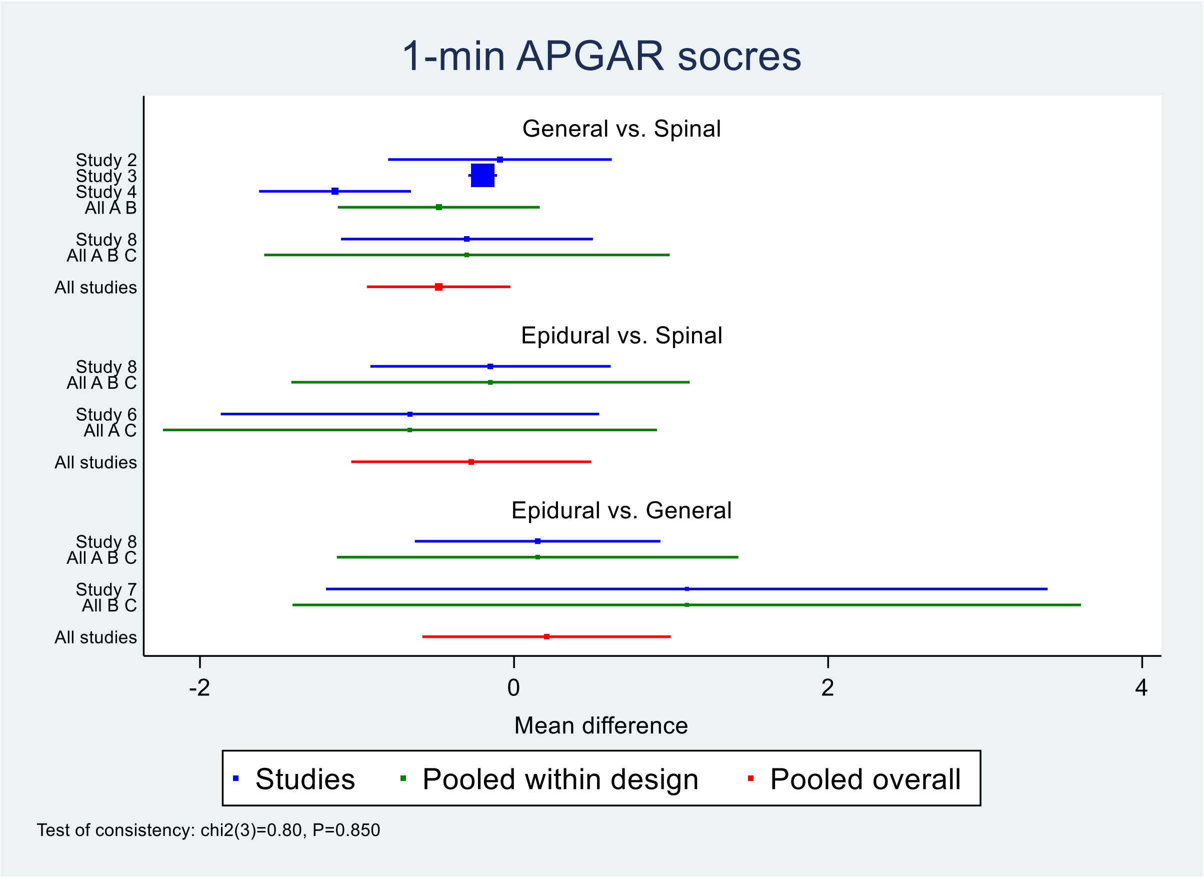

Appendix 17. Publication bias test of 5-min APGAR scores

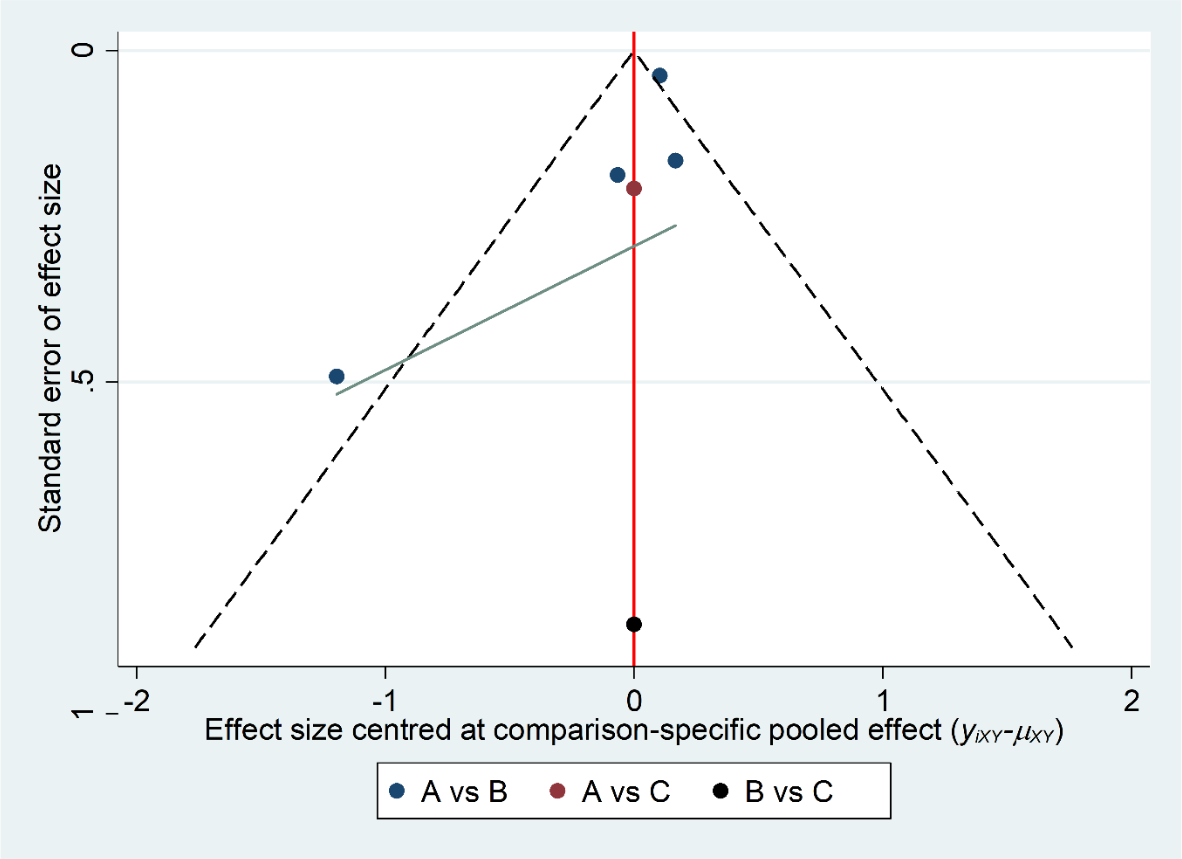

Egger's test for small-study effects:

Number of studies = 6 Root MSE = 1.127

Std_Eff	Coef.	Std. Err.	t	P>|t|	[95% Conf. Interval]	
slope	.1453288	.0529696	2.74	0.052	-.0017383	.2923959
bias	-.9503172	.614008	-1.55	0.197	-2.655077	.7544424

Test of H0: no small-study effects P = 0.197

Appendix 18. Publication bias test of 1-min APGAR scores

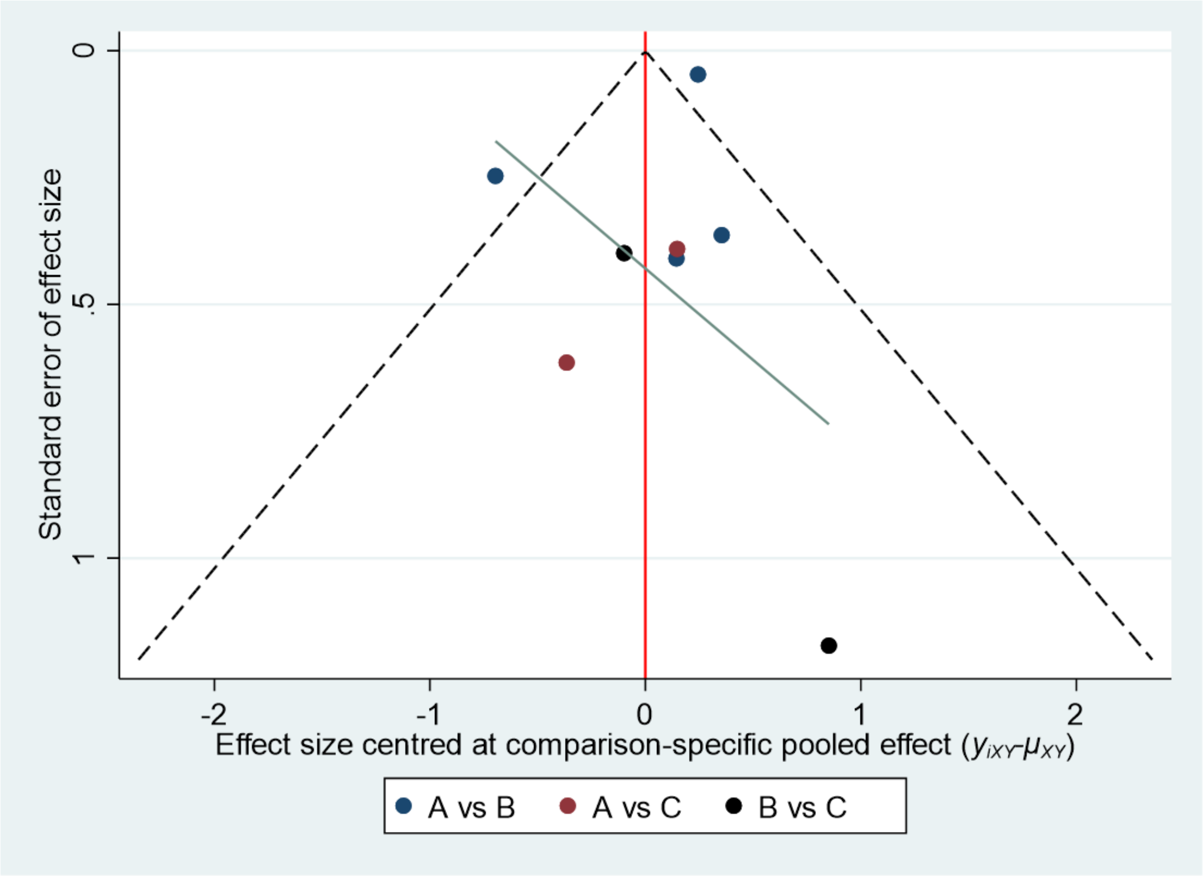

Number of studies = 8 Root MSE = 1.474

Std_Eff	Coef.	Std. Err.	t	P>|t|	[95% Conf. Interval]	
slope	.2637685	.0822443	3.21	0.018	.0625238	.4650131
bias	-.759859	.652469	-1.16	0.288	-2.356393	.8366752

Test of H0: no small-study effects P = 0.288

Appendix 19. Network plot of neonatal resuscitation

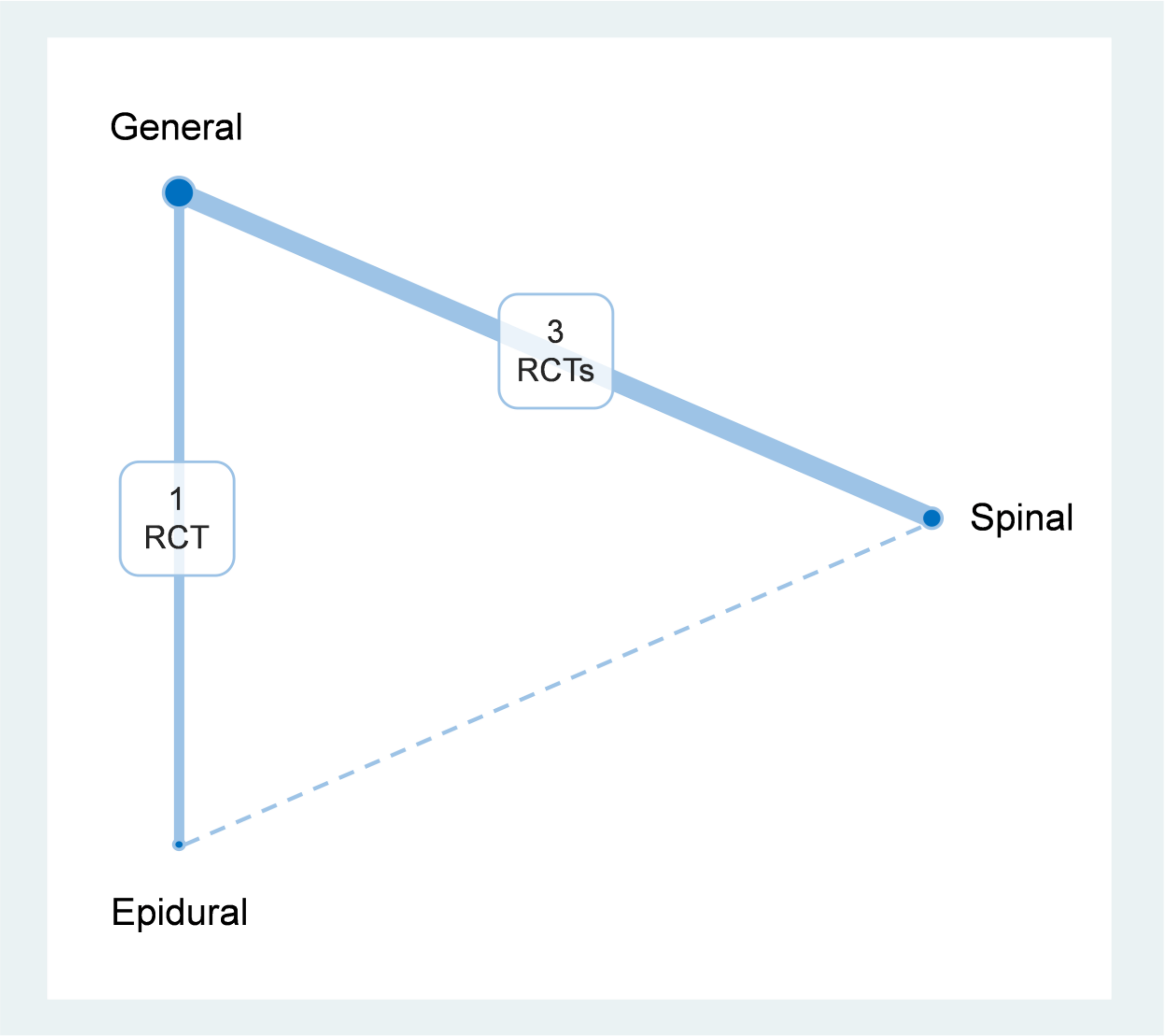

Appendix 20. Forest plot of neonatal resuscitations

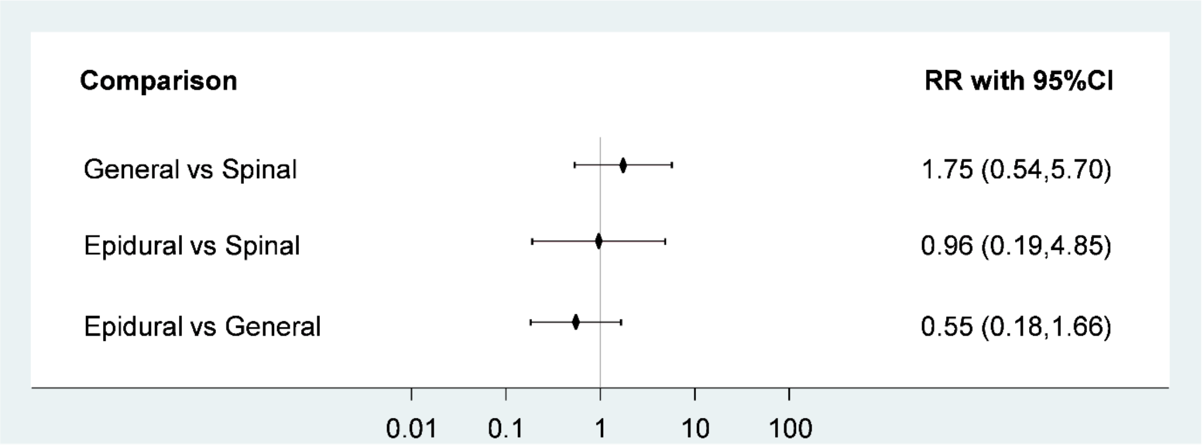

Appendix 21. Publication bias test of neonatal resuscitations

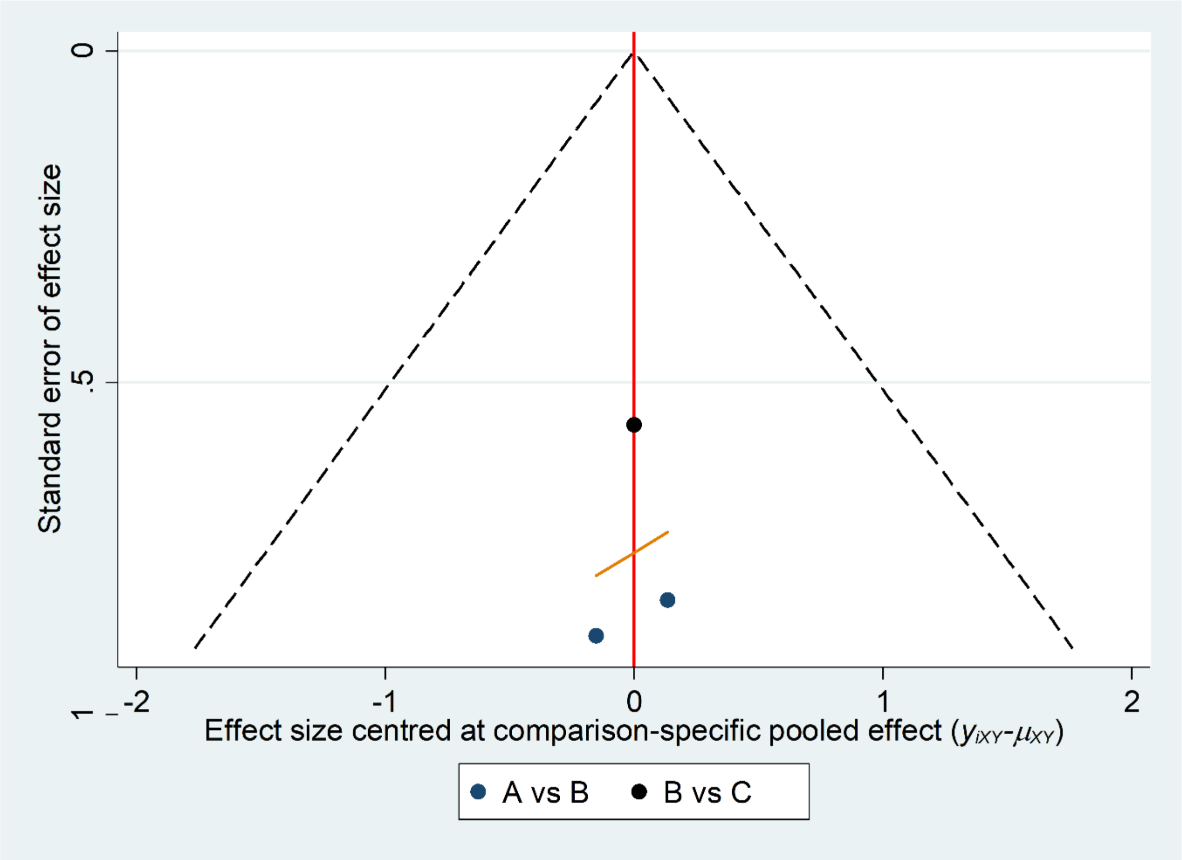

Egger's test for small-study effects:

Number of studies = 3 Root MSE = .2362

Std_Eff	Coef.	Std. Err.	t	P>|t|	[95% Conf.	Interval]
slope	.0600847	.4771852	0.13	0.920	-6.003129	6.123298
bias	-.0860865	.6693515	-0.13	0.919	-8.591003	8.41883

Test of H0: no small-study effects P = 0.919

Appendix 22. Search strategy

Primary search strategy

#1. preeclampsia
#2. pregnancy toxemia
#3. gestosis
#4. eph
#5. #1 OR #2 OR #3 OR #4
#6. general anesthesia
#7. epidural anesthesia
#8. spinal anesthesia
#9. anesthesia
#10. lumbar anesthesia
#11. combined spinal epidural anesthesia
#12. analgesia
#13. #6 OR #7 OR #8 OR #9 OR #10 OR #11 OR #12
#14. #5 AND #13
No filter.

Syntax in Embase :

('preeclampsia'/exp OR 'pre eclampsia' OR 'pre-eclampsia' OR 'pre-eclamptic toxaemia' OR 'pre-eclamptic toxemia' OR 'preclampsia' OR 'preeclampsia' OR 'preeclamptic toxaemia' OR 'preeclamptic toxemia' OR 'toxaemia, preeclamptic' OR 'toxemia, preeclamptic' OR 'pregnancy toxemia'/exp OR 'eph gestosis' OR 'eclamptic toxaemia' OR 'eclamptic toxemia' OR 'eclamptogenic toxaemia' OR 'eclamptogenic toxemia' OR 'eph syndrome' OR 'gestational toxaemia' OR 'gestational toxemia' OR 'gestational toxicosis' OR 'gestosis' OR 'gestosis, eph' OR 'hep syndrome' OR 'pregnancy toxaemia' OR 'pregnancy toxaemias' OR 'pregnancy toxemia' OR 'pregnancy toxemias' OR 'pregnancy toxicosis' OR 'toxaemia gravidum' OR 'toxemia gravidum' OR 'toxemic pregnancy' OR 'toxicosis gravidarum') AND ('anesthesiological procedure'/exp OR 'anaesthesia and analgesia' OR 'anesthesia and analgesia' OR 'anesthesiological procedure' OR 'anesthesiological techniques' OR 'spinal anesthesia'/exp OR 'anaesthesia, spinal' OR 'anaesthesia, subarachnoid' OR 'analgesia spinal' OR 'anesthesia, spinal' OR 'anesthesia, subarachnoid' OR 'complete spinal block' OR 'lumbar anaesthesia' OR 'lumbar anesthesia' OR 'lumbar extradural blockade' OR 'partial spinal block' OR 'rachianaesthesia' OR 'rachianesthesia' OR 'spinal anaesthesia' OR 'spinal anaesthetic' OR 'spinal analgesia' OR 'spinal anesthesia' OR 'spinal block' OR 'spinal cord anaesthesia' OR 'spinal cord anesthesia' OR 'subarachnoid anaesthesia' OR 'subarachnoid anesthesia' OR 'subarachnoid block' OR 'subarachnoidal anaesthesia' OR 'subarachnoidal anesthesia' OR 'epidural anesthesia'/exp OR 'anaesthesia, epidural' OR 'anaesthesia, peridural' OR 'anesthesia, epidural' OR 'anesthesia, peridural' OR 'dural blocking' OR 'epidural anaesthesia' OR 'epidural anaesthetic agent' OR 'epidural anesthesia' OR 'epidural anesthetic agent' OR 'epidural block' OR 'epidural blockade' OR 'extradural anaesthesia' OR 'extradural anesthesia' OR 'extradural block' OR 'lumbar epidural anaesthesia' OR 'lumbar epidural anesthesia' OR 'lumbar peridural anaesthesia' OR 'lumbar peridural anesthesia' OR 'lumbar peridural block' OR 'peridural anaesthesia' OR 'peridural anesthesia' OR 'peridural block' OR 'peridural blocking' OR 'general anesthesia'/exp OR 'anaesthesia, general' OR 'anesthesia, general' OR 'general anaesthesia' OR 'general anesthesia' OR 'combined spinal epidural anesthesia'/exp OR 'anesthetic agent'/exp OR 'anaesthetic' OR 'anaesthetic agent' OR 'anaesthetic drug' OR 'anaesthetics' OR 'anaesthetics, combined' OR 'anaesthetics, dissociative' OR 'anaesthetics, general' OR 'anesthetic' OR 'anesthetic agent' OR 'anesthetic drug' OR 'anesthetics' OR 'anesthetics, combined' OR 'anesthetics, dissociative' OR 'anesthetics, general' OR 'general anaesthetic' OR 'general anaesthetic agent' OR 'general anesthetic' OR 'general anesthetic agent' OR 'preanaesthetics' OR 'preanesthetics' OR 'analgesic agent'/exp OR 'agent, analgesic' OR 'analgesic' OR 'analgesic agent' OR 'analgesic drug' OR 'analgesic evaluation' OR 'analgesic tv 393' OR 'analgesics' OR 'analgesics, non narcotic' OR 'analgesics, non-narcotic' OR 'analgesics, topical' OR 'analgetic' OR 'analgetic agent' OR 'analgetic drug' OR 'anodyne' OR 'antalgic agent' OR 'non narcotic analgesic' OR 'non narcotic analgesic agent' OR 'nonnarcotic analgesic' OR 'nonnarcotic analgesic agent' OR 'epidural analgesia'/exp OR 'analgesia, epidural' OR 'epidural analgesia' OR 'extradural analgesia' OR 'peridural analgesia') AND ('cesarean section'/exp OR 'birth, abdominal operation' OR 'birth, caesarean' OR 'caesarean birth' OR 'caesarean section' OR 'caesarian birth' OR 'caesarian section' OR 'cesarean delivery' OR 'cesarean section' OR 'cesarian section' OR 'elective repeat cesarean section' OR 'fetectomy' OR 'repeated cesarotomy' OR 'sectio caesarea')

Syntax in Cochrane Database of Systematic Reviews and Cochrane Central Register of Controlled Trials :

(preeclampsia OR pregnancy toxemia OR gestosis OR eph) AND (general anesthesia OR epidural anesthesia OR spinal anesthesia OR anesthesia OR lumbar anesthesia OR combined spinal epidural anesthesia OR analgesia)

Syntax in New PubMed :

((((("pre-eclampsia"[MeSH Terms] OR "pre eclampsia"[All Fields]) OR "preeclampsia"[All Fields]) OR (((("pregnancy toxaemia"[All Fields] OR "pre-eclampsia"[MeSH Terms]) OR "pre eclampsia"[All Fields]) OR ("pregnancy"[All Fields] AND "toxemia"[All Fields])) OR "pregnancy toxemia"[All Fields])) OR "gestosis"[All Fields]) OR "eph"[All Fields]) AND ((((((((("general anaesthesia"[All Fields] OR "anesthesia, general"[MeSH Terms]) OR ("anesthesia"[All Fields] AND "general"[All Fields])) OR "general anesthesia"[All Fields]) OR ("general"[All Fields] AND "anesthesia"[All Fields])) OR (((("epidural anaesthesia"[All Fields] OR "anesthesia, epidural"[MeSH Terms]) OR ("anesthesia"[All Fields] AND "epidural"[All Fields])) OR "epidural anesthesia"[All Fields]) OR ("epidural"[All Fields] AND "anesthesia"[All Fields]))) OR (((("spinal anaesthesia"[All Fields] OR "anesthesia, spinal"[MeSH Terms]) OR ("anesthesia"[All Fields] AND "spinal"[All Fields])) OR "spinal anesthesia"[All Fields]) OR ("spinal"[All Fields] AND "anesthesia"[All Fields]))) OR (((("anaesthesia"[All Fields] OR "anesthesia"[MeSH Terms]) OR "anesthesia"[All Fields]) OR "anaesthesias"[All Fields]) OR "anesthesias"[All Fields])) OR (((((((("lumbarised"[All Fields] OR "lumbarization"[All Fields]) OR "lumbarized"[All Fields]) OR "lumbars"[All Fields]) OR "lumbosacral region"[MeSH Terms]) OR ("lumbosacral"[All Fields] AND "region"[All Fields])) OR "lumbosacral region"[All Fields]) OR "lumbar"[All Fields]) AND (((("anaesthesia"[All Fields] OR "anesthesia"[MeSH Terms]) OR "anesthesia"[All Fields]) OR "anaesthesias"[All Fields]) OR "anesthesias"[All Fields]))) OR (((((((((("combinable"[All Fields] OR "combinated"[All Fields]) OR "combination"[All Fields]) OR "combinational"[All Fields]) OR "combinations"[All Fields]) OR "combinative"[All Fields]) OR "combine"[All Fields]) OR "combined"[All Fields]) OR "combines"[All Fields]) OR "combining"[All Fields]) AND (((("spinal"[All Fields] OR "spinalization"[All Fields]) OR "spinalized"[All Fields]) OR "spinally"[All Fields]) OR "spinals"[All Fields]) AND (((("epidural anaesthesia"[All Fields] OR "anesthesia, epidural"[MeSH Terms]) OR ("anesthesia"[All Fields] AND "epidural"[All Fields])) OR "epidural anesthesia"[All Fields]) OR ("epidural"[All Fields] AND "anesthesia"[All Fields])))) OR (("analgesia"[MeSH Terms] OR "analgesia"[All Fields]) OR "analgesias"[All Fields]))
